# Phylogeographic Analysis of *Blastomyces dermatitidis* and *Blastomyces gilchristii* Reveals an Association with North American Freshwater Drainage Basins

**DOI:** 10.1371/journal.pone.0159396

**Published:** 2016-07-18

**Authors:** Lisa R. McTaggart, Elizabeth M. Brown, Susan E. Richardson

**Affiliations:** 1 Public Health Laboratories Toronto, Public Health Ontario, Toronto, Ontario, Canada; 2 Department of Laboratory Medicine and Pathobiology, University of Toronto, Toronto, Ontario, Canada; 3 Division of Microbiology, Department of Paediatric Laboratory Medicine, The Hospital for Sick Children, Toronto, Ontario, Canada; University of Arkansas, UNITED STATES

## Abstract

*Blastomyces dermatitidis* and *Blastomyces gilchristii* are dimorphic fungal pathogens that cause serious pulmonary and systemic infections in humans. Although their natural habitat is in the environment, little is known about their specific ecologic niche(s). Here, we analyzed 25 microsatellite loci from 169 strains collected from various regions throughout their known endemic range in North America, representing the largest and most geographically diverse collection of isolates studied to date. Genetic analysis of multilocus microsatellite data divided the strains into four populations of *B*. *dermatitidis* and four populations of *B*. *gilchristii*. *B*. *dermatitidis* isolates were recovered from areas throughout North America, while the *B*. *gilchristii* strains were restricted to Canada and some northern US states. Furthermore, the populations of both species were associated with major freshwater drainage basins. The four *B*. *dermatitidis* populations were partitioned among (1) the Nelson River drainage basin, (2) the St. Lawrence River and northeast Atlantic Ocean Seaboard drainage basins, (3) the Mississippi River System drainage basin, and (4) the Gulf of Mexico Seaboard and southeast Atlantic Ocean Seaboard drainage basins. A similar partitioning of the *B*. *gilchristii* populations was observed among the more northerly drainage basins only. These associations suggest that the ecologic niche where the sexual reproduction, growth, and dispersal of *B*. *dermatitidis* and *B*. *gilchristii* occur is intimately linked to freshwater systems. For most populations, sexual reproduction was rare enough to produce significant linkage disequilibrium among loci but frequent enough that mating-type idiomorphic ratios were not skewed from 1:1. Furthermore, the evolutionary divergence of *B*. *dermatitidis* and *B*. *gilchristii* was estimated at 1.9 MYA during the Pleistocene epoch. We suggest that repeated glaciations during the Pleistocene period and resulting biotic refugia may have provided the impetus for speciation as theorized for other species associated with temperate freshwater systems.

## Introduction

Most fungi that are pathogenic to humans reside naturally in the environment with occasional transmission to humans or other animals. Many fungi, especially the dimorphic fungi, display a limited and distinct phylogeographic distribution, suggesting that growth and persistence in the environment is linked to specific biogeographic and ecological factors [1–14]. For example, *Coccidioides immitis* is localized primarily to the San Joaquin Valley of California, while *Coccidioides posadasii* has a wider biogeographical distribution including southwestern United States, southern California, Mexico, and South America [2]. Yet, little is known about the specific ecologic niche of human fungal pathogens that are acquired from the environment. An understanding of the ecological factors that favor growth, reproduction, and dispersal would potentially provide a means of predicting and controlling human acquisition of infection. Fortunately, highly discriminatory genetic typing methods such as multilocus microsatellite typing can help infer ecological factors controlling the reproduction and propagation of fungal pathogens in the environment, as we demonstrate here for the systemic fungal pathogens in the genus *Blastomyces*.

The genus *Blastomyces* represents two species of thermally dimorphic fungi: *Blastomyces dermatitidis* and *Blastomyces gilchristii* [15]. In North America, *Blastomyces* species are considered endemic to the midwestern, southeastern, and south central United States bordering the Ohio and Mississippi River valleys, and the Canadian provinces and US states bordering the Great Lakes and St. Lawrence River [16]. There are reports of blastomycosis from other countries, specifically China, Zimbabwe, South Africa, and India [17–20]; however, endemicity in these regions is somewhat dubious. Several strains of African origin were shown by DNA melt curve analysis to represent a separate taxon, possibly *Emmonsia* spp. [21], and case reports from China and India involved patients with a recent travel history to the endemic regions in North American [18,19]. Still, at least five older cases from 1982–1998 involving two humans, a dog, and two bats from India appear to be autochthonous [19]. Thus, North America represents the primary region of endemicity for *Blastomyces* spp. Although direct isolation of the fungus from the environment has rarely been successful [22], a variety of ecological factors favoring growth have been postulated based on human and canine case studies [23–36].

In a recent study, multilocus sequence typing (MLST) was used to demonstrate the presence of a cryptic species within what was previously considered a single species *Blastomyces dermatitidis* [15]. The cryptic species, named *Blastomyces gilchristii*, was found to be reproductively and genetically isolated from *B*. *dermatitidis* and appeared to have an overlapping, although perhaps smaller, geographic distribution compared to *B*. *dermatitidis* [15]. Similar groupings have been noted by others [37], who also suggested that differences in clinical manifestations may exist between the two groups [38]. More recently, whole genome sequencing of select *B*. *dermatitidis* (ER-3, ATCC 18188 and ATCC 26199) and *B*. *gichristii* (SLH14081) strains has provided additional evidence for the delineation of these two species [39].

The goal of this study was to use multilocus microsatellite typing (MLMT) to elucidate the population structures of *B*. *dermatitidis* and *B*. *gilchristii* from, what is to our knowledge, the largest and most geographically diverse collection of isolates studied to date. In total, four populations of *B*. *dermatitidis* and four populations of *B*. *gilchristii* were detected, with the geographic distribution of the populations partitioned among the major freshwater drainage basins of North America. The results of our analysis indicate that the reproduction, growth, and dispersal of *Blastomyces* spp. are intimately linked to freshwater systems, confirming what was previously suspected based on epidemiological investigations of blastomycosis outbreaks [33]. The study demonstrates that discriminatory genetic analyses, such as MLMT, are powerful methods for elucidating information about the population structure and ecology of human pathogenic fungi in a manner that is relevant to understanding and possibly controlling disease acquisition.

## Results

### Population structure of *B*. *dermatitidis* and *B*. *gilchristii*

All 25 MLMT loci were amplified for 139 of 169 (82.2%) isolates. Of the remaining 30 isolates, 24 of 25 loci were typed for 27 (16%) isolates, 23 of 25 loci were typed for 2 isolates (1.2%), and a single isolate had 22 of 25 loci typed (0.6%) (S1 Table). The inability to type all *Blastomyces* isolates using this scheme has been noted previously [37].

**Fig 1:**
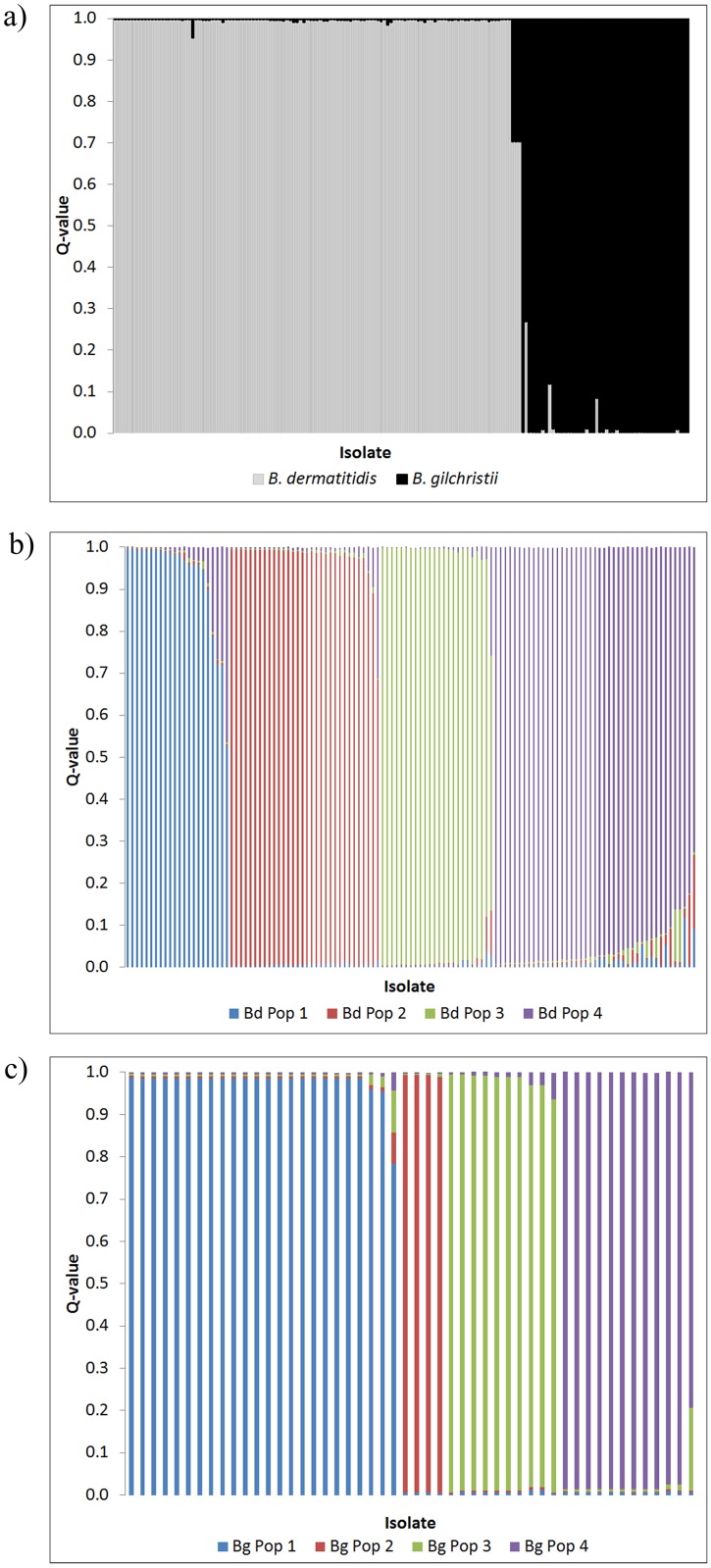
Plot of individual STRUCTURE Q-values (averaged across all 8 iterations) demonstrating the proportion of an isolate’s genotype that belongs to a) *B*. *dermatitidis* and *B*. *gilchristii* b) populations 1–4 of *B*. *dermatitidis* or c) populations 1–4 of *B*. *gilchristii*.

Initial analysis of the microsatellite data with the STRUCTURE software suggested two biologically meaningful genetic clusters with a large peak in the ΔK profile (S1a Fig). Plots of the individual STRUCTURE Q-values (averaged across all 8 iterations), which show the posterior mean estimates of the proportion of each isolate’s genome inherited from ancestors of each group, indicate little admixture between the two groups (Fig 1a). The most probable ancestor analysis by STRUCTURE, which assigns each isolate to a cluster, indicated that one group contained only isolates previously identified as *B*. *dermatitidis* by MLST (n = 24) while the second group contained only *B*. *gilchristii* isolates (n = 26) [15]. Therefore, the remaining isolates assigned to group 1 and group 2 were identified as *B*. *dermatitidis* or *B*. *gilchristii*, respectively. Using multilocus sequence typing (MLST) data [15], *BEAST estimated the divergence time of *B*. *dermatitidis* and *B*. *gilchristii* at 1.9 MYA (95% HPD 0.5–4.4 MYA).

STRUCTURE analysis of the *B*. *dermatitidis* and the *B*. *gilchristii* microsatellite data analyzed independently suggested that each species contained four populations (S1b Fig). Plots of the individual STRUCTURE Q-values indicate some admixture between the populations of each group (Fig 1b and 1c). The most probably ancestor algorithm was used to assign each isolate to one of *B*. *dermatitidis* populations 1–4 or *B*. *gilchristii* populations 1–4.

*B*. *dermatitidis* and its populations exhibited much higher levels of intra-population genetic diversity than *B*. *gilchristii*, as indicated by the higher values for the mean effective number of alleles (N_e_) and mean haploid genetic diversity (H_e_) (Table 1). Although the number of isolates sampled for *B*. *dermatitidis* was more than twice that of *B*. *gilchristii*, this likely had little effect on the N_e_ and H_e_ values since 15–20 isolates is considered sufficient for accurately estimating heterozygosity (i.e. genetic diversity) [40]. *B*. *gilchristii* exhibited low intra-population genetic diversity, with only 21 unique microsatellite types represented among the 50 isolates.

**Table 1:** Population genetic analysis and mating-type idiomorph determination of each population of *Blastomyces* spp.

Population	Drainage Basin	n^1^	MT^2^	N_a_^3^	N_e_^4^	H_e_^5^	P_a_^6^	Mating-Type Idiomorph			
								*α*-box	HMG	ND^7^	χ^2^, p-value (clone-corrected)
***B*. *dermatitidis***		119	111	14.280± 1.011	7.254± 0.598	0.831± 0.018	12.600±1.023	56 (47.1%)	57 (47.9%)	7 (5.9%)	χ^2^ = 0.009,p = 0.924 (χ^2^ = 0.01,p = 0.920)
Pop 1	Nelson River	20	19	4.840± 0.411	2.860± 0.232	0.586± 0.037	0.760± 0.202	8 (40%)	11 (55%)	1 (5%)	χ^2^ = 0.474,p = 0.491 (χ^2^ = 0.222,p = 0.638)
Pop 2	St. Lawrence River	32	29	6.720± 0.453	4.024± 0.347	0.718± 0.020	1.560± 0.413	15 (46.9%)	14 (43.8%)	3 (9.4%)	χ^2^ = 0.034,p = 0.854 (χ^2^ = 0.154,p = 0.695)
Pop 3	South-eastern US	24	23	4.640± 0.299	3.142± 0.215	0.633± 0.033	0.640± 0.199	12 (50%)	10 (41.7%)	2 (8.3%)	χ^2^ = 0.182,p = 0.670 (χ^2^ = 0.429,p = 0.512)
Pop 4	Mississippi River	43	40	10.520±0.731	6.143± 0.582	0.799± 0.020	3.320± 0.320	21 (48.8%)	22 (51.2%)	0 (0%)	χ^2^ = 0.023,p = 0.879 (χ^2^ = 0.100,p = 0.752)
***B*. *gilchristii***		50	21	2.400± 0.311	1.435± 0.119	0.216± 0.046	0.720± 0.204	18 (36.0%)	30 (60.0%)	2 (4.0%)	χ^2^ = 3.0,p = 0.083 (χ^2^ = 1.0,p = 0.317)
Pop 1	Nelson River	24	5	1.200± 0.082	1.049± 0.035	0.032± 0.019	0.040± 0.040	11 (45.8%)	12 (50%)	1 (4.2%)	χ^2^ = 0.043,p = 0.836 (χ^2^ = 0.143,p = 0.705)
Pop 2	Mississippi River	4	2	1.080± 0.055	1.048± 0.033	0.030± 0.021	0.080± 0.055	2 (50%)	2 (50%)	0 (0%)	χ^2^ = 0.0,p = 1.0 (χ^2^ = 0.333,p = 0.564)
Pop 3	St. Lawrence River	10	9	1.720± 0.220	1.400± 0.163	0.181± 0.044	0.280± 0.136	3 (30%)	6 (60%)	1 (10%)	χ^2^ = 1.0,p = 0.317 (χ^2^ = 1.0,p = 0.317)
Pop 4	St. Lawrence River	12	5	1.160± 0.075	1.045± 0.023	0.034± 0.017	0.000± 0.000	2 (16.7%)	10 (83.3%)	0 (0%)	χ^2^ = 5.333,p = 0.021 (χ^2^ = 0.667,p = 0.414)

^1^n = number of samples

^2^MT = number of microsatellite types

^3^N_a_ = mean number of alleles

^4^N_e_ = mean effective number of alleles

^5^H_e_ = mean haploid genetic diversity

^6^P_a_ = mean number of private alleles

^7^Mating-type idiomorph not detected by PCR amplification

The genetic distance between *B*. *dermatitidis* and *B*. *gilchristii* was 1.369 with higher pairwise genetic distances between populations of different species than populations of the same species (Table 2). Interestingly, *B*. *dermatitidis* population 4 isolates were genetically more similar to the other three *B*. *dermatitidis* populations than any of the other three populations were to each other. In comparison, the pairwise genetic distance values of the *B*. *gilchristii* populations were quite low, which is likely due to the low genetic diversity within the species (Table 2). F_ST_ values were used to ascertain the proportion of genetic variance among geographic regions relative to the total variance [5]. F_ST_ values close to zero indicate that genetic variation is shared within and between the populations, whereas higher F_ST_ values indicate that more genetic variation occurs between populations compared to those within populations [5]. Our F_ST_ values indicate that the populations were significantly differentiated (*p* < 0.001) from one another (Table 2).

**Table 2:** Pairwise population F_ST_ values (below the diagonal) with significant values (*p*<0.001) in bold. Pairwise Nei’s genetic distances above the diagonal.

	***Bd***^1^ **Pop1**	***Bd* Pop2**	***Bd* Pop3**	***Bd* Pop4**	***Bg***^2^ **Pop1**	***Bg* Pop2**	***Bg* Pop3**	***Bg* Pop4**
***Bd* Pop1**		1.013	1.315	0.716	2.011	1.882	1.650	1.782
***Bd* Pop2**	**0.2120**		0.892	0.502	1.462	1.511	1.283	1.386
***Bd* Pop3**	**0.2856**	**0.1910**		0.778	2.351	2.333	1.641	1.870
***Bd* Pop4**	**0.1548**	**0.0879**	**0.1499**		1.779	1.642	1.591	1.764
***Bg* Pop1**	**—**	**—**	**—**	**—**		0.274	0.285	0.279
***Bg* Pop2**	**—**	**—**	**—**	**—**	**0.8708**		0.487	0.433
***Bg* Pop3**	**—**	**—**	**—**	**—**	**0.7313**	**0.6647**		0.183
***Bg* Pop4**	**—**	**—**	**—**	**—**	**0.8710**	**0.8976**	**0.5597**	

^*1*^*Bd* represents B. *dermatitidis*

^*2*^*Bg* represents *B*. *gilchristii*

The microsatellite dataset was also analyzed by TreeMix v1.1 to detect any potential migration events between the populations [41]. We identified two migration events, one between *B*. *gilchristii* populations 1 and 4 (*p* = 7.3x10^-5^) and one between *B*. *gilchristii* populations 1 and 2 (*p* = 0.017) (S2 Fig), which correlate with the comparably lower genetic distance values (Table 2).

### Geographic distribution of *B*. *dermatitidis* and *B*. *gilchristii* populations

**Fig 2:**
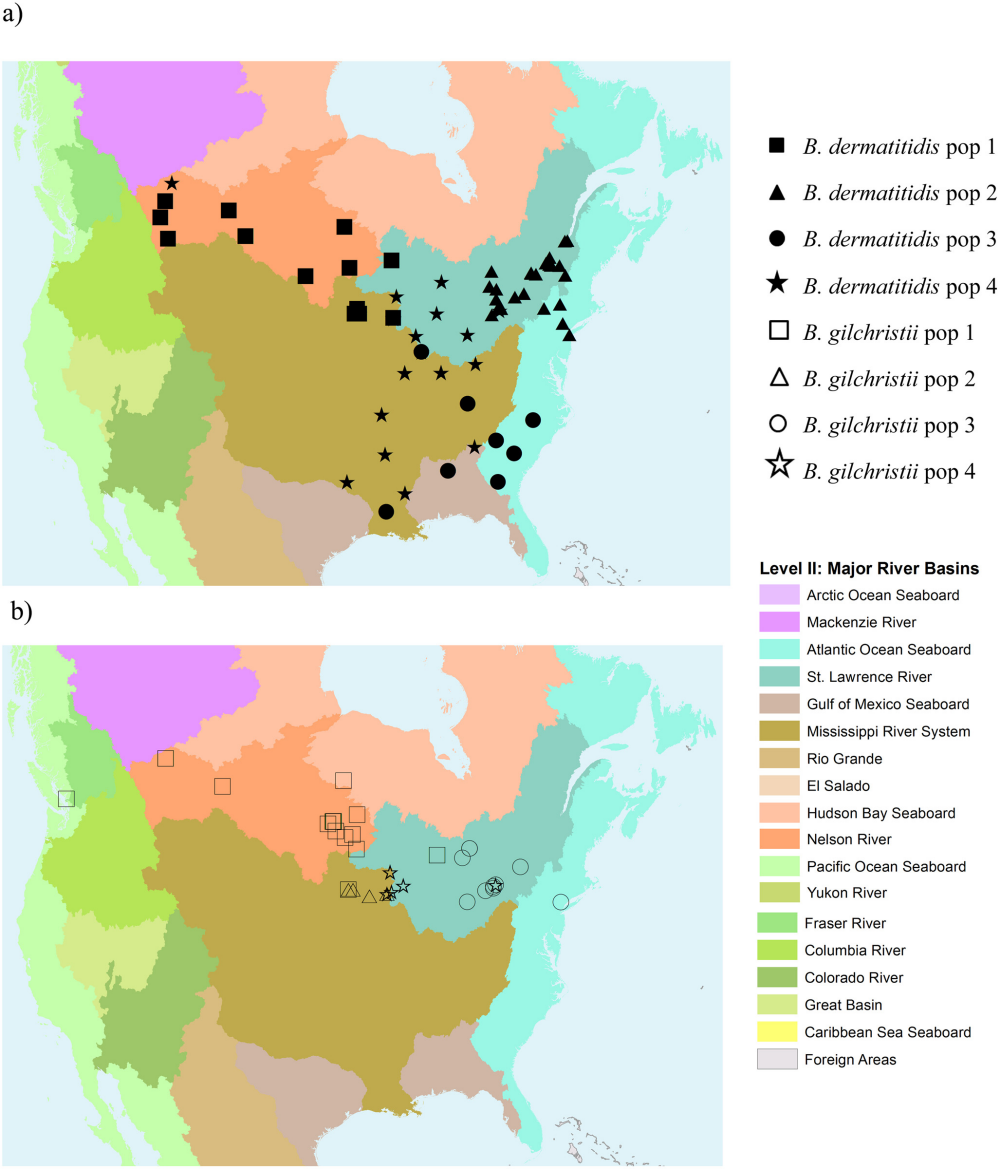
Maps of North America displaying the geographic location of isolates of (a) *B*. *dermatitidis* and (b) *B*. *gilchristii* in relation to the major freshwater drainage basins. Isolates are coded based on the population to which STRUCTURE assigned them. Maps were constructed using ArcGIS 10.2.1 software (ESRI, Toronto, ON) and map files from the Commission for Environmental Cooperation [42].

Analysis of geographic location revealed that *B*. *dermatitidis* isolates were present throughout North America from all sampled regions. Conversely, *B*. *gilchristii* isolates were recovered only from select Canadian provinces and northern US states, specifically, Alberta, Saskatchewan, British Columbia, Ontario, New York, Minnesota, and Wisconsin. Furthermore, the various populations of *B*. *gilchristii* and *B*. *dermatitidis* appeared to be partitioned according to the major North American river drainage basins. *B*. *dermatitidis* population 1 isolates were sampled primarily from the region of the Nelson River drainage basin (covering northern Minnesota, northeastern North Dakota, a small portion of northwestern Ontario, and the southern half of Manitoba, Saskatchewan, and Alberta). Population 2 isolates were from the St. Lawrence River drainage basin (covering portions of Quebec, Vermont, New York, Ontario, Michigan, and Wisconsin surrounding the Great Lakes and the St. Lawrence Seaway) and the northeast Atlantic Ocean Seaboard drainage basin (covering most of New York). Population 3 isolates were largely derived from the Gulf of Mexico Seaboard drainage basin (Alabama) and the southeast Atlantic Ocean Seaboard drainage basin (covering Georgia, South Carolina, and North Carolina). Population 4 isolates were primarily obtained from the Mississippi River System drainage basin (covering part or all of Montana, Wyoming, Colorado, Arizona, North Dakota, South Dakota, Nebraska, Kansas, Oklahoma, Minnesota, Wisconsin, Iowa, Missouri, Arkansas, Mississippi, Louisiana, Illinois, Indiana, Ohio, Pennsylvania, West Virginia, Kentucky, Tennessee, Georgia, Alabama, and North Carolina). (Table 1, S2 Table, Fig 2a). *B*. *gilchristii* population 1 isolates were sampled primarily from regions of the Nelson River drainage basin, similar to *B*. *dermatitidis* population 1. *B*. *gilchristii* population 2 contained only four isolates that were derived from the region that could be considered the northern-most part of the Mississippi River System drainage basin (specifically the Mississippi River (trunk) sub-basin (Fig 2b)). *B*. *gilchristii* population 3 contained isolates from the St. Lawrence River drainage basin and northeast Atlantic Ocean Seaboard drainage basin. *B*. *gilchristii* population 4 isolates were almost exclusively from Wisconsin, hailing from both the Mississippi River System drainage basin and St. Lawrence River drainage basin (Table 1, S2 Table, Fig 2b).

**Fig 3:**
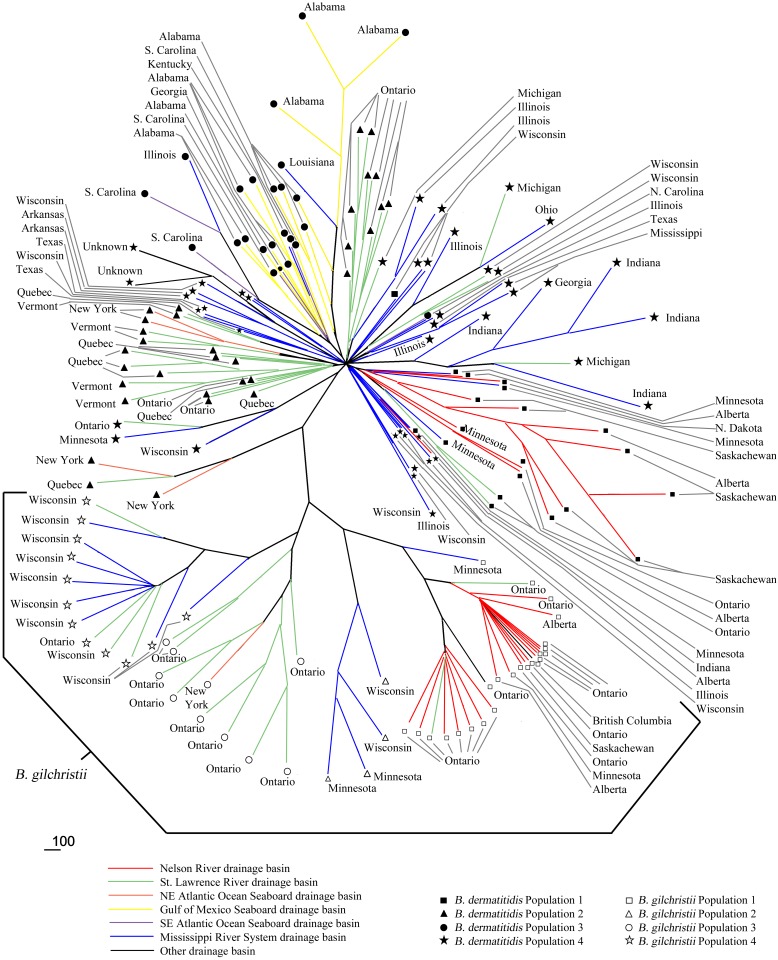
Majority rule (extended) consensus tree constructed from 500 bootstrap replicates using the neighbour-joining method with Nei’s genetic distances calculated from fragment sizes of 25 microsatellites of 169 isolates of *B*. *dermatitidis* and *B*. *gilchristii*. Branches are colour-coded by drainage basin. Branches are labelled with the state or province from which the isolate was derived and the population to which STRUCTURE assigned them. Bar represents Nei’s genetic distance = 100.

A consensus neighbour-joining phylogenetic tree confirmed not only the deep genetic divide between the *B*. *dermatitidis* and *B*. *gilchristii* isolates, but also the genetic similarity of isolates associated with the same drainage basin (Fig 3). A few exceptions were observed. Of note, three Minnesota isolates of *B*. *dermatitidis*, from Hennepin and Anoka counties, clustered with population 1 even though their geographic location was in the Mississippi River System drainage basin. Likewise one isolate each from Illinois (Gu), Kentucky (K966), and Louisiana (Ro) clustered with population 3 but were geographically associated with the Mississippi River System drainage basin. Two isolates from Thunder Bay, Ontario located in the St. Lawrence River drainage basin grouped with the population 1 isolates, most of which were from the adjoining Nelson River drainage basin. One Ontario isolate, two Wisconsin (Milwaukee) isolates, and three Michigan isolates from the St. Lawrence River drainage basin clustered with the population 4 isolates, along with one canine isolate from Edmonton (Nelson River drainage basin) and one N. Carolina isolate (SE Atlantic Ocean Seaboard drainage basin). Among the *B*. *gilchristii* isolates, one isolate from Minnesota (Hennepin County) and two isolates from Ontario (Sault Ste. Marie) clustered with population 1, but were geographically located in the Mississippi River System drainage basin and the St. Lawrence River drainage basin, respectively. Also, one isolate from British Columbia (Fraser River drainage basin) and one isolate from North Spirit Lake, Ontario (Hudson Bay Seaboard drainage basin) clustered with the population 1 isolates (S2 Table). Inconsistencies were noted between the STRUCTURE assignments and the consensus NJ tree, namely ATCC-28306 and F2012034121 (population 1 isolates that cluster with population 4) and DI 13–61 (a population 3 isolate that clusters with population 4). These inconsistencies were likely due to different method algorithms coupled with higher levels of admixture noted in F2012034121 and DI 13–61 and a unique MLMT profile for ATCC-28306.

To further investigate the association between genetic relatedness and drainage basin localization, a series of distance based redundancy analyses (db-RDA) and partial db-RDAs were performed. The analyses suggested that for both species, genetic distance between isolates was significantly correlated with (constrained by) drainage basin (*B*. *dermatitidis* pseudo-*F* = 5.5916, *p* < 0.001; *B*. *gilchristii* pseudo-*F* = 13.233, *p* < 0.005 respectively) even when controlling for geographical distance (*B*. *dermatitidis* pseudo-*F* = 2.7827, *p* <0.001, *B*. *gilchristii* pseudo-*F* = 7.0482, *p* < 0.005 respectively). For *B*. *dermatitidis*, drainage basin explained 11.4% of the variation in genetic distance between isolates. Of this, 5.0% was due to drainage basin alone while 6.4% was due to a joint effect of drainage basin and geographic distance, since the two are not unrelated explanatory factors. An additional 1.5% was explained by geographic distance alone. For *B*. *gilchristii*, 23.2% of the variation in genetic distance was attributed to drainage basin, with 17.0% due to a joint effect of drainage basin and geographic distance. An additional 1.3% was explained by geographic distance alone. The remaining 87.1% and 75.5% of the variation in genetic distance of *B*. *dermatitidis* and *B*. *gilchristii* respectively was unexplained and may be attributed to other factors not examined in this study.

### Linkage disequilibrium and recombination analysis

Like many fungi, *Blastomyces* reproduces both asexually and sexually. Therefore, a series of analyses evaluating linkage disequlibrium and mating-type idiomorph were conducted to assess the relative proportion of sexual and asexual reproduction in *Blastomyces*. The level of multilocus linkage disequilibrium in the dataset was high, with significant r_d_ values both with and without clone correction, (Table 3). Only the largely homogeneous *B*. *gilchristii* populations 1 and 4 exhibited non-significant r_d_ values when analyzed independently, indicating random mixis (Table 3). Additionally, the parsimony tree permutation length test (PTPLT) showed that the observed trees of the complete dataset partitioned into either two (*B*. *dermatitidis* and *B*. *gilchristii*, *p*<0.001) or eight populations (*B*. *dermatitidis* populations 1–4 and *B*. *gilchristii* populations 1–4 *p*<0.001), the *B*. *dermatitidis* dataset partitioned into four populations (*p*<0.001), and the *B*. *gilchristii* dataset partitioned into four populations (*p* = 0.009) were significantly shorter than trees generated following 1000 random shufflings of the data (S3 Fig). Similar results were observed with clone-corrected datasets, with *p*-values of *p*<0.001, *p*<0.001, *p*<0.001, and *p* = 0.018, respectively (S3 Fig). Thus taken together, the significant linkage disequilibrium and the PTPLT results suggest a lack of complete mixis within the four *B*. *dermatitidis* and at least one of the four *B*. *gilchristii* populations. However, when the seven supercontigs that contained multiple microsatellite loci were analyzed independently for linkage disequilibrium, mixis was detected for supercontigs 1 and 3 for both species and for supercontigs 1, 2, 4, and 20 for the *B*. *gilchristii* clone-corrected dataset (Table 4), which suggests sexual recombination.

**Table 3:** Linkage disequilibrium r_d_ values for populations *of B*. *dermatitidis* and *B*. *gilchristii* both with and without clone correction.

Population (Data partition)	r_d_ (*p*-value)	Clone corrected r_d_ (*p*-value)
***B*. *dermatitidis* (4 pops)**	0.054 (<0.001)	0.048 (<0.001)
Pop1	0.115 (<0.001)	0.0940 (<0.001)
Pop2	0.0898 (<0.001)	0.068 (<0.001)
Pop3	0.122 (<0.001)	0.115 (<0.001)
Pop4	0.0582 (<0.001)	0.0447 (<0.001)
***B*. *gilchristii* (4 pops)**	0.194 (<0.001)	0.114 (<0.001)
Pop1	0.048 (0.174)	-0.050 (0.770)
Pop2	n/a^a^	n/a^a^
Pop3	0.254 (<0.001)	0.234 (<0.001)
Pop4	0.114 (0.174)	0.0139 (0.528)

^a^ There were not enough isolates in *B*. *gilchristii* population 2 to accurately estimate r_d_.

**Table 4:** Linkage disequilibrum r_d_ values of microsatellite loci located on supercontigs of *B*. *dermatitidis* or *B*. *gilchristii* with data partitioned into 4 populations for each species both with and without clone correction.

Supercontig	Loci	r_d_ (*p*-value)	Clone corrected r_d_ (*p*-value)
*B*. *dermatitidis* (4 pops)			
Supercontig 2	MLMT 1,2,3	0.0891 (<0.001)	0.0848 (<0.001)
Supercontig 4	MLMT 4,5,6,7	0.0981 (<0.001)	0.1001 (<0.001)
Supercontig 3	MLMT 8,9,10	-0.0102 (0.989)	-0.004 (0.966)
Supercontig 1	MLMT 13,15	0.0748 (0.075)	0.0662 (0.065)
Supercontig 20	MLMT 18,19,20	0.0746 (<0.001)	0.067 (<0.001)
Supercontig 60	MLMT 23,24	0.0959 (<0.001)	0.0969 (0.001)
Supercontig 16	MLMT 26,27	0.119 (0.001)	0.121 (<0.001)
*B*. *gilchristii* (4 pops)			
Supercontig 2	MLMT 1,2,3	0.089 (<0.001)	0.0612 (0.273)
Supercontig 4	MLMT 4,5,6,7	0.098 (<0.001)	0.167 (0.164)
Supercontig 3	MLMT 8,9,10	-0.0102 (0.989)	n/a^a^
Supercontig 1	MLMT 13,15	0.0748 (0.075)	0.055 (1)
Supercontig 20	MLMT 18,19,20	0.0746 (<0.001)	0.091 (0.221)
Supercontig 60	MLMT 23,24	0.0985 (<0.001)	n/a^a^
Supercontig 16	MLMT 26,27	0.118 (0.001)	n/a^a^

^a^There were not enough isolates to accurately estimate r_d_.

Mating-type idiomorphic ratios were examined as an indirect measure of the level of sex and recombination within the *Blastomyces* species [5]. The mating-type idiomorphic ratios did not differ significantly from the 1:1 ratio expected for a sexual population, with the exception of *B*. *gilchristii* population 4 (Table 1). However, following clone correction, the mating-type idiomorphic ratio of this population was also not significantly different from 1:1 (Table 1). Furthermore, a single pair of *B*. *dermatitidis* clones (11PHO547 (HMG) and SF12545/2009 (α Box)) and three sets of *B*. *gilchristii* clones (set 1: 12PHO936, TB00002/2008, TB00019/2005, TB00037/2008, TB00017/2006 (HMG), and TB00016/2005, TB00025/2005 (α Box); set 2: 12PHO859, F2013001127, M09MY002766, M10MY005485, M11MY006516, TB00018/2005, TB00029/2006, TB00040/2005 (α Box), and M08MY007367, TB00018/2006, TB00022/2006, TB00023/2005, TB00038/2005 (HMG); and set 3: 590, 600, 641, ATCC 60636, ATCC 62583, ATCC MYA-2585 (HMG), and ATCC 62541 (α Box)) each displayed identical MLMT profiles but different mating-type idiomorphs (S1 and S2 Tables).

## Discussion

Microsatellite typing of 169 North American strains of *Blastomyces* confirmed the presence of two species, *B*. *dermatitidis* and *B*. *gilchristii*, and revealed the presence of several differentiated populations within each species. Furthermore, the geographic localization of each population was generally partitioned among the major freshwater drainage basins of North America. This study represents a discriminatory population genetic analysis of the largest and most geographically diverse collection of *Blastomyces* isolates examined to date, including strains from various regions throughout their known endemic range in North America.

Although previously known as *B*. *dermatitidis*, the presence of two distinct species, *B*. *dermatitidis* and *B*. *gilchristii*, was recently documented based on multilocus sequence typing and recombination analysis [15]. The microsatellite typing and STRUCTURE analysis performed in this study confirms the presence of two markedly distinct genetic groups, which correlate perfectly with the MLST species delineation in a subset of 50 strains. It also corroborates the findings of other researchers who have also noted two distinct genetic groups based on microsatellite typing and whole genome sequencing [37,39]. Little is known about the phenotypic differences between *B*. *dermatitidis* and *B*. *gilchristii*, however, preliminary studies have suggested that there may be differences in the clinical spectrum of disease between the two species [38]. Based on the geographic analysis in the current study, *B*. *dermatitidis* appears to be present throughout North America from the southeastern United States to Western Canada. Conversely, *B*. *gilchristii* appears to be limited to a northern range, present primarily in central Canada and a few of the north central US states bordering Canada. *B*. *gilchristii* was found in Ontario, Saskatchewan, Alberta, Minnesota, and Wisconsin; however it was rarely detected among strains isolated from states and provinces bordering the eastern seaboard (i.e. Quebec, New York, and Vermont). While more extensive sampling may reveal the presence of *B*. *gilchristii* in additional Canadian provinces and northern US states, the level of sampling done in this study allows us to exclude central and southern US from the endemic range of *B*. *gilchristii*.

The entire set of 25 microsatellite loci were typed from 82.2% of strains with 22–24 loci typed from the remaining strains. The inability to type all *Blastomyces* isolates using this scheme has been noted previously [37]. Although we report a higher percentage of isolates that failed to type at one or more loci, this is probably due to the fact that a more diverse collection of isolates was employed in this study. Nevertheless, other studies involving microsatellite typing of fungi typically employ fewer loci, between 9 and 21 [2,13,14,43–46], suggesting that the current dataset is sufficient to discriminate the population structure of *Blastomyces* despite the missing data.

The isolates tested in this study were partitioned into four genetically distinct populations of *B*. *dermatitidis* and four genetically distinct populations of *B*. *gilchristii*, with the overall geographic localization of the different populations delineated by the major freshwater drainage basins of North America. Although these observations are similar to the geographic delineation of strains described previously [15], these populations were empirically determined and represent a refinement of the previously hypothesized groups. Among the *B*. *dermatitidis* isolates, population 1 was associated with the Nelson River drainage basin; population 2 with the St. Lawrence River drainage basin and the northeast portion of the Atlantic Ocean Seaboard drainage basin; population 3 with the Gulf of Mexico Seaboard and southeast portion of the Atlantic Ocean Seaboard drainage basin; and population 4 with the Mississippi River System drainage basin. For populations 2 and 3, a 1:1 assignment of population to drainage basin was not observed; rather a single population detected by STRUCTURE analysis of the microsatellite data was distributed over two adjacently located drainage basins. It is possible that additional subpopulations also delineated by drainage basin do exist but were undetectable by MLMT. Alternatively, additional ecological factors or mechanisms of dispersal, other than freshwater drainage (i.e. wind dispersal), may also be influencing the geographic distribution of these populations and provide an explanation for the distribution of these populations across adjacent drainage basins.

*B*. *gilchristii* was also delineated into four populations with one population associated with the Nelson River drainage basin, a second population associated with what is presumably the northern-most tip of the Mississippi River System drainage basin, and a third population associated with the St. Lawrence River and northeast Atlantic Ocean Seaboard drainage basins. With the exception of a single isolate from Toronto, Ontario, the fourth population consisted entirely of isolates from Eagle River, Oconto Falls, and Tomorrow River, Wisconsin. Although the Wisconsin isolates in this population were located no more than 115 miles (190 km) apart [47], they were split between the Mississippi River System and St. Lawrence River drainage basins. While freshwater drainage appears to be a major determinate of the geographic localization of *Blastomyces*, the *B*. *gilchristii* population 4 isolates are genetically highly similar and yet are partitioned among two major freshwater drainage basins. These observations suggest that alternate climatic or environmental determinants or other mechanisms of dispersal (i.e. wind dispersal) impacted the geographic distribution of these populations on a more localized scale. Furthermore, there is evidence that the *B*. *gilchristii* populations are not genetically isolated with migration detected from the more northerly population 1 to the more southerly populations 2 and 4 (S2 Fig).

Distance-based redundancy analysis indicated that localization to drainage basin was a significant explanatory factor accounting for the genetic structure (analyzed as genetic distance) of *B*. *dermatitidis* (*p* < 0.001) and *B*. *gilchristii* (*p* < 0.005) isolates. In fact, for both species, drainage basin localization was a more important explanatory factor than geographic distance, accounting for 5.0% vs. 1.5% of the variation in genetic distance for *B*. *dermatitidis* and 6.1% vs. 1.3% for *B*. *gilchristii*. However, the variation partitioning of genetic distance not explained by drainage basin localization and/or geographic position was substantial, 87.1% for *B*. *dermatitidis* and 75.5% for *B*. *gilchristii*. Thus, many other factors may be responsible for the variation in genetic distance observed between isolates, not the least of which would be the stochastic nature of microsatellite mutations which are generally considered to be selectively neutral [48]. Overall, these findings provide statistical support to the hypothesis that the population genetic structure of *Blastomyces* is influenced by drainage basin localization.

Although the link between genetic similarity represented by the clustering of isolates into populations and geographic localization to a drainage basin is readily apparent, exceptions within our dataset do exist. As noted above, other ecological variables and modes of dispersal are likely also shaping the geographic localization of genetically distinct populations. Another potentially significant cause of these exceptions is travel, since the travel histories of the patients from whom the isolates were derived were unknown. Due to the lengthy incubation period of blastomycosis (1–4 months) [32,33], a person can acquire the infection in one location but travel to another location before the symptoms develop and the disease is diagnosed. It is also possible that a person may develop blastomycosis in a rural area of one drainage basin, but travel to an urban area in another drainage basin for diagnosis and treatment. This may be particularly relevant for certain Ontario and Minnesota strains, where it is plausible that the infection could have been acquired in rural areas associated with the Nelson River drainage basin, but diagnosed in an urban area (e.g. Minneapolis, Minnesota, or Thunder Bay or Sault Ste. Marie, Ontario) located in the Mississippi or St. Lawrence River drainage basins, respectively. Thus, environmental isolates would be ideal for this type of geographic analysis, however, they are extremely difficult to obtain [22]. Finally, although most of the isolates in this study are recent (acquired within the last 3–10 years) a handful of strains were isolated up to 50 years ago. Thus, there are temporal differences in addition to geographic differences between the isolates, which could be influencing the results.

The association between geographic localization to a drainage basin and genetic population structure implies that the reproduction, growth, and dispersal of both *B*. *dermatitidis* and *B*. *gilchristii* are intimately linked to freshwater systems. The ecologic niche of *Blastomyces* has long been an active area of investigation, in large part due to the difficulty in isolating strains directly from the environment, especially soil [22]. There are fewer than 25 reported isolations of this fungus from the environment, representing a less than 1% success rate, using a variety of techniques, including animal inoculation and a variety of artificial media [49,50]. Only 45% of these isolations have come from soil or soil-containing specimens [32,33,51–55]. In fact, soil appears to have an inhibitory effect on the survival of the fungus in either its mycelial or yeast form [56], whereas woody plant material appears to support its growth best in vitro, especially when combined with soil extract [57]. Based on epidemiological features of canine and human blastomycosis in certain geographic locales, a variety of environmental features have been postulated to facilitate the growth of *Blastomyces*, including sandy acidic soil, fluctuations in soil moisture levels, waterways at low elevations, decaying organic matter, high humidity (approaching 100%), coniferous forested regions, and proximity to bodies of water [23–34,36]. Proximity to freshwater has been emphasized repeatedly by these studies and ecologic niche modeling which noted that proximity to waterways was a distinguishing characteristic of areas that had the highest predicted occurrence of blastomycosis [26,35]. Experimentally, water has been shown to be critical to the efficient dispersal of the infectious conidia of *Blastomyces* [58]. Both the 1984 [32] and 2006 [22] Wisconsin outbreaks were associated with warm, rainy and/or windy weather prior to each outbreak. Together these studies suggest that the fungus may require soil/organic nutrients for mycelial growth and reproduction prior to conidial liberation and dispersal by freshwater. Our phylogeographic analysis provides evidence that freshwater systems, probably through their conidial liberating function, serve to define the distribution of this fungus along major waterways and its genetic separation and differentiation over time.

Like *Blastomyces* spp., microsatellite and/or sequence typing of other fungi has revealed that most fungi exhibit genetically divergent populations localized to specific geographic regions [1–14]. Two notable exceptions are *Aspergillus fumigatus* and *Penicillium chrysogenum* which show world-wide dispersal with no correlation between genotype and geographic location [59–61]. Furthermore, population genetic structure has been used to infer the mechanism of dispersal of various fungal species, including dispersion by host species [3,5,6,45], environmental factors such as seawater and extreme weather [12,62], and human transportation [8,14,44]. While many fungi exhibit a worldwide distribution, *Blastomyces* species exhibit a relatively restricted endemic range, primarily central and eastern North America for *B*. *dermatitidis* and the north central US and Canada for *B*. *gilchristii*. However, if indeed freshwater basins or their shorelines create the optimal environmental locale for reproduction and dissemination; this may explain why the endemic region of these two fungi is relatively restricted. Unlike other fungi where population structure and dispersal is linked to a host species (either animal hosts or plants transported by humans) or large-scale climactic (wind or seawater) factors, the opportunities for large-scale spread of *Blastomyces* species via freshwater may simply have been limited. Alternatively, *Blastomyces* may simply be very fastidious, with the resulting ecologic niche capable of supporting growth and reproduction mainly limited to the freshwater systems of North America.

Like many fungi, *Blastomyces* is capable of clonal propagation as well as sexual reproduction [63]. As well, genetic recombination indicative of sexual reproduction was previously demonstrated by a variety of tests (PTPLT, linkage disequilibrium I_A_ and r_d_ values, split decomposition analysis, Phi test, and 4-gametes tests) performed on seven nuclear gene sequences of the two species [15]. Moreover, whole genome sequencing has identified reduced levels of synteny in the GC-poor regions of select *B*. *gilchristii* and *B*. *dermatitidis* strains, effectively preventing opportunities for meiotic recombination between species and providing additional evidence for their delineation [39]. Among fungi, the relative contributions of the two modes of growth, clonal propagation and sexual reproduction, differ and will have an impact on population genetic structure [4,5,43]. For our microsatellite dataset, the level of linkage disequilibrium within most populations was significant (with the exception of *B*. *gilchristii* populations 1 and 4), and PTPLT analysis suggested that the populations were not freely recombining. The significant level of linkage disequilibrium is contrasted with the low (non-significant) level of linkage disequilibrium previously detected in the multilocus nuclear gene sequence dataset [15]. This is probably due to the different levels of heterozygosity in the datasets, since the detection of linkage depends on polymorphisms [59]. Despite the substantial linkage disequilibrium, we did detect recombination within each species in at least two of the supercontigs containing multiple microsatellite loci, indicating that sexual reproduction does occur [59]. Furthermore, MLMT clones with different mating-type idiomorphs were detected among both the *B*. *dermatitidis* and the *B*. *gilchristii* isolates, which provides explicit evidence of sexual recombination on the smallest possible scale [5]. The mating-type idiomorphic ratios of the populations of either species (clone-corrected data) was not significantly different from the 1:1 ratio expected for fungi undergoing regular sexual reproduction [59]. Thus, it is probable that in addition to clonal propagation, both *B*. *dermatitidis* and *B*. *gilchristii* undergo regular sexual reproduction and recombination at a rate sufficient to maintain a 1:1 ratio of mating-types idiomorphs, but rare enough that significant linkage disequilibrium exists within each population.

Based on Bayesian analysis of nucleotide sequences, we estimate that *B*. *dermatitidis* and *B*. *gilchristii* diverged 1.9 MYA during the Pleistocene epoch. Spanning 2.58 to 0.011 MYA, the Pleistocene epoch was characterized by a series of glaciations events that advanced to envelop large portions of North America, reaching as far south as Iowa, Illinois, Indiana, and Ohio, and then receded [64]. The repeated glaciations caused unparalleled alterations to the freshwater environment, destroying some habitats and creating new systems of lakes and rivers [64]. While most temperate species experienced a reduction and fragmentation of their habitat during this period, populations of freshwater organisms were particularly affected, as the displacement and geographic isolation of subsets of populations created an opportunity for allopatric speciation events [64,65]. When the glaciers retreated, large proglacial lakes formed which could have enabled the widespread distribution of aquatic species [64]. In North America, the Pleistocene glaciations are considered a “speciation pump” and are responsible for reproductive isolation and allopatric speciation of several freshwater fish species. Genetic diversity and intraspecific divergence was greater for freshwater fish species in southern non-glaciated regions compared to northern glaciated regions [64,66]. Given the association of *B*. *dermatitidis* and *B*. *gilchristii* with the freshwater drainage basins in North America and estimated time of divergence, it is possible that their speciation was caused by the Pleistocene glaciations. Allopatric speciation could have occurred through displacement of local population subsets to different refugia, via the proposed conidial-liberating function of water on the glacial fronts. Similarly, one population subset may have remained frozen in glacial ice for thousands of years while another subset evolved at lower latitudes. The meltwater produced during the glacial retreat could have mixed the two species to create the overlapping geographic distributions observed today. As temperatures warmed during the last glacial maximum (18 000–21 000 years ago), populations within each species may have expanded their historical geographic distribution from isolated refugia as additional landscapes became suitable [65]. Like freshwater aquatic species [66], we noted a deeper tree topology, greater genetic diversity, and greater intra-specific divergence for *B*. *dermatitidis* whose geographic range was largely non-glaciated (i.e. the southern Mississippi River basin and southeastern United States) as compared to *B*. *gilchristii*, whose geographic range was entirely glaciated.

In conclusion, microsatellite typing coupled with phylogeographic analysis elucidated the population structure of the environmental fungi and serious human pathogens *B*. *dermatitidis* and *B*. *gilchristii*. This analysis suggested that the major freshwater drainage basins of North America are an essential component of their ecologic niches and provides a plausible mechanism for the dispersal, speciation, and population structure of North American *Blastomyces*. Further investigation using this knowledge will provide a greater understanding of the ecology of these important fungi; thereby controlling exposure where possible and facilitating the rapid recognition of outbreaks when they occur, leading to prompt public health responses and appropriate treatment.

## Materials and Methods

### Strains and culture conditions

One hundred and sixty-nine strains of *Blastomyces* spp. were collected from various regions of North America (S2 Table). Strains were isolated from human (n = 164), canine (n = 1), and environmental (n = 4) sources between 1963 and 2013. Fifty of the strains included in this study were previously examined by MLST and identified as *B*. *dermatitidis* or *B*. *gilchristii* [15]. Strains from Canada included 44 strains from Ontario obtained from clinical samples submitted to the Mycology laboratory at Public Health Ontario (Toronto, ON, Canada) between 2005 and 2013; 1 Ontario strain from the America Type Culture Collection (Manassa, VA, USA); 4 Alberta strains, 5 Saskatchewan strains, and 1 British Columbian strain from the Alberta Health Services, University of Alberta Hospital (Edmonton, AB, Canada); 10 Quebec strains from the Laboratoire de santé publique du Québec (Sainte-Anne-de-Bellevue, Québec, Canada); and 5 strains (3 from Saskatchewan and 2 from Alberta) from the University of Alberta Microfungus and Herbarium (Edmonton, AB, Canada). From the United States, 30 strains (8 from Illinois, 7 from Wisconsin, 5 from Vermont, 3 from Texas, 3 from Missouri, 2 from Arkansas, 1 from North Carolina, and 1 from Ohio) were obtained from the University of Texas Fungus Testing Laboratory (San Antonio, TX, USA); 10 strains (8 from Wisconsin, 1 from Georgia, and 1 from South Carolina) from the America Type Culture Collection (Manassa, VA, USA); 19 strains (7 from Wisconsin, 3 from South Carolina, 2 from Mississippi, 2 from Illinois, 2 of unknown origin, 1 from Kentucky, 1 from Georgia, and 1 from Louisiana) from David Stevens, Santa Clara Valley Medical Center (San Jose, CA, USA); 12 strains (10 from Minnesota, 1 from North Dakota, and 1 from Wisconsin) were obtained from the Minnesota Department of Health (St. Paul, MN, USA); 3 Michigan strains from the Michigan Department of Community Health (Lansing MI, USA); 5 Indiana strains from the Indiana State Department of Health (Indianapolis, IN, USA); 15 Alabama stains from the University of Alabama (Birmingham, Alabama); and 5 New York strains from the Wadsworth Center (Albany, NY, USA). Strains were identified as *Blastomyces* spp. by the referring facility using one or more conventional direct detection methods i.e. microscopy, culture identification, mould-yeast phase conversion, AccuProbe *Blastomyces dermatitidis* culture identification test (Gen-Probe, San Diego, CA), or PCR and sequencing. Upon receipt at PHO, strains were cultured as mold on potato dextrose agar (BD, Mississauga, ON) incubated at 25°C and subsequently subcultured to D-media [67] and incubated at 37°C for 1–3 weeks to convert to the yeast phase.

This study was approved by the Ethics Review Board at Public Health Ontario. *Blastomyces* specimens were collected from sources listed in the methods section, and the data were analyzed anonymously.

### Multilocus microsatellite typing

DNA was isolated from D-media yeast cultures using the Norgen Fungi/Yeast Genomic DNA Isolation Kit (Norgen Biotek, Thorold, ON, Canada). Primers and PCR reactions described by Meece et al. (2011) [37] were used to amplify 25 microsatellite loci from each strain. Loci 17 and 21 were omitted from our dataset due to inconsistent amplification among strains. Furthermore, reverse primers for loci 3, 7, and 10 were modified to include 5’ tails (gggatgcTGCTGATTCAGACGGTGAAG, gggatgcTAGATTTCGAGCCCAGCATT, and gggatgcCAAAATGGGAAAGGAAAGCA, respectively) to eliminate ambiguity due to the inconsistent incorporation of +A artifacts during PCR amplification. PCR products were diluted 1 in 10 in water before mixing 0.5 μl of the PCR product with 0.5 μl of GeneScan-500 ROX size standard (Life Technologies, Carlsbad, CA, USA) and 9.0 μl of Hi-Di formamide (Life Technologies). Samples were denatured at 95°C for 3 minutes and cooled immediately on ice prior to fragment analysis using a 50-cm capillary array on a Genetic Analyzer 3730xl (Life Technologies). Results were analyzed using GeneMapper v4.0 (LifeTechnologies) with microsatellite fragments scored by fragment size (S1 Table).

### Phylogenetic and population genetic analysis

The number of populations represented by the microsatellite data was assessed using the Bayesian clustering method implemented in the software STRUCTURE v2.3.4 [68–70]. Using the admixture model with correlated frequencies, we tested the probability of 1 to 10 clusters for the entire dataset, the *B*. *dermatitidis* isolates only, and the *B*. *gilchristii* isolates only. Each model was simulated 8 times for 500,000 simulations with a burn-in of 100,000. The number of clusters was determined using the ad hoc statistic delta K (ΔK) [71], with the ancestral population identity of each strain determined from the most probable ancestor algorithm (Q-output) of STRUCTURE.

MLMT genotypes were analyzed using GenAlEx 6.5 [72] to determine the allelic diversity, genetic diversity, and Nei’s genetic distance among all populations. F_ST_ values (Weir’s formulation based on haplotype frequencies [73]) were calculated using the Microsatellite analyzer software (MSA) v4.05 [74].

Microsatellite data were analyzed using TreeMix v1.1 [41] to build a maximum likelihood tree and infer admixture events with sample size correction turned off. Based on an initial tree, *B*. *gilchristii* population 1 was chosen as the outgroup. Migration events were added (m = 2) until the *p*-value exceeded 0.05.

A neighbor-joining (NJ) phylogenetic tree was constructed using Nei’s genetic distance matrix [75] calculated by MSA. Although all loci were previously reported to contain dinucleotide repeats [37], we noted a range of fragment sizes for most microsatellite loci suggesting the presence of indels within the flanking regions. Therefore, we chose to use Nei’s genetic distance calculation to measure genetic distance, since this it is appropriate for obtaining a correct tree topology from complex microsatellite data where an infinite-allele model is appropriate [76]. A Majority Rule (extended) tree was constructed from 500 bootstrap replications of the neighbour-joining algorithm using Phylip v3.7a [77] with visual presentation in TreeView v1.6.6 [78].

In order to assess the correlation between genetic distance and geographic distance, pairwise matrices based on Nei’s genetic distance [75] were calculated and constructed for the *B*. *dermatitidis* and the *B*. *gilchristii* isolates using MSA. *B*. *dermatitidis* isolates SACS and SACR were omitted from this dataset due to uncertainty in geographic origin [79]. Depending on the information available, the geographic location of each isolate was coded as the latitude and longitude of the approximate centre of the city, county, or state/province (S2 Table) with two exceptions: three isolates from Texas were assigned a location in the eastern portion of the state near the Mississippi River and 1 isolate from Alberta was placed in the south-central portion of the province in keeping with what is known about the endemic range of *Blastomyces* [16]. Additionally, each isolate was categorized into a major North American river drainage basin based on their geographic location (S2 Table). The major North American river drainage basins considered were the: Nelson River drainage basin, Hudson Bay Seaboard drainage basin, Fraser River drainage basin, St. Lawrence River drainage basin, Mississippi River System drainage basin (which included the Arkansas/Red River, Mississippi River (trunk), Missouri River, and Ohio River sub-basins), Gulf of Mexico Seaboard drainage basin, northeast Atlantic Ocean Seaboard drainage basin, and southeast Atlantic Ocean drainage basin. No isolates were assigned to the remaining North American major river drainage basins.

Distance based redundancy analysis (db-RDA) [80] was used to explore the relationship between the response matrix (genetic distance) and an explanatory matrix (geographic distance and/or drainage basin). Using R software [81], the latitude and longitude geographic coordinates were transformed into a 2-dimensional orthogonal system expressed in kilometers using the geoXY function of the SoDA package. Using the vegan package, a principal coordinate analysis (PCoA) of the genetic distance matrix was computed, correcting for negative eigenvalues. All principal coordinates were retained and tested against the explanatory variables of geographic distance and/or drainage basin (treated as categorical data) using db-RDA and partial db-RDA. The significance of the db-RDA was tested by comparing the true value of the *pseudo-F* test statistic to those of 1000 random permutations of the data with significance assigned if the proportion of the permuted values (*p* value) that was equal to or smaller than the true value was less than or equal to *α* = 0.05. Adjusted R^2^ values were computed for each test and the partition of variance attributable to each of the explanatory variables was calculated [80].

The distribution of isolates belonging to each *B*. *dermatitidis* and *B*. *gilchristii* population was mapped across North America using ArcGIS 10.2.1 software (ESRI, Toronto, ON) and maps imported from the Commission for Environmental Cooperation [42].

### Recombination and linkage disequilibrium analysis

Linkage disequilibrium (r_d_) values were calculated using Multilocus v1.3b [82] for the complete data sets and the clone-corrected data sets of *B*. *dermatitidis* and *B*. *gilchristii* divided into four populations each (*B*. *dermatitidis* populations 1–4 and *B*. *gilchristii* populations 1–4) (Table 1). Additionally, each population was assessed independently, with the exception of *B*. *gilchristii* population 2 as there were not enough isolates to accurately estimate r_d_. Statistical significance of the r_d_ values was determined by comparing them to the distribution of r_d_ values from 1000 randomized permutations of the data (*p* ≤ 0.05). Furthermore, linkage disequilibrium (r_d_) was assessed individually for each of the supercontigs of the ATCC 26199 genome sequence by the Broad Institute [83]. The following linkages were applied: MLMT-13 and MLMT 15 (supercontig 1), MLMT-1, MLMT-2, MLMT-3 (supercontig 2), MLMT-8, MLMT-9, MLMT-10 (supercontig 3), MLMT-4, MLMT-5, MLMT-6, MLMT-7 (supercontig 4), MLMT-18, MLMT-19 and MLMT-20 (supercontig 20), MLMT-23 and MLMT-24 (supercontig 60), MLMT-26 and MLMT-27 (supercontig 16). The parsimony tree permutation length test (PTPLT) was performed to detect random mating using PAUP 4.0 BETA [84] using files generated by Multilocus v3.1 as previously described [15].

The mating-type idiomorph of each isolate was determined as previously described [15,37] with frequencies compared to the null hypothesis ratio of 1:1 using chi square tests (*p* ≤ 0.05).

### Divergence time estimation

The estimated time of divergence of *B*. *dermatitidis* and *B*. *gilchristii* was estimated using the *BEAST extension of BEAST v1.8.0 [85,86]. We used partial sequences of 7 nuclear genes (*arf6*, *chs2*, *drk1*, *fads*, *its-2*, *pyrF*, and *tub1*) of 40 *B*. *dermatitidis* isolates and 40 *B*. *gilchristii* isolates, previously published [15]. The substitution rate of *its-2* was fixed at 8.3 x10−^4^ substitutions per site per million years as previously estimated for *B*. *dermatitidis* [87]. The evolutionary rates of the remaining genes were estimated relative to the fixed *its-2* substitution rate (*arf* 6 9.828 x10^-4^, *chs2* 9.537 x10^-4^, *drk1* 1.292 x10^-3^, *fads* 4.622 x10^-4^, *opd* 1.371 x10^-3^, *tub1* 1.668 x10^-3^) [85,86]. An uncorrelated relaxed exponential clock with an exponential prior distribution and a mean of 1.0 x10−^3^ substitutions per site per million years [87] was implemented for all genes. The clock, substitution, and tree models were unlinked as appropriate for nuclear genes capable of recombination. The HKY substitution model was chosen for all genes with en estimated base frequency but without gamma site heterogeneity or invariant sites. The Yule speciation model was employed with a random starting tree for each gene. Two independent, identical *BEAST analyses were run each for 100 million generations with trees and parameters sampled every 10,000 generations. The two runs were examined in Tracer v1.6 [88] to confirm that all parameters had converged on the same stationary distribution with effective sample sizes >200. The two runs were combined in LogCombiner v1.8.0 with burn-in values of 2500 for each tree file, thus discarding the first 25% of samples, and annotated in TreeAnnotator v1.8.0 to generate the maximum clade credibility tree.

## Supporting Information

S1 Fig Delta K (ΔK) values calculated from the posterior probability values generated by STRUCTURE analysis of populations K = 1–10 for (a) all isolates and (b) *B*. *dermatitidis* and *B*. *gilchristii* isolates. Where Delta K (ΔK) is equal to, ΔK = m(|L”(K)|)/s[L(K)].
(TIF)

S2 Fig Maximum-likelihood tree inferred by TreeMix v1.1 for populations of *B*. *dermatitidis* (*Bd*) and *B*. *gilchristii* (*Bg*), allowing for 2 migration events. Migration arrows are coloured according to their weight. Horizontal branch lengths are proportional to the amount of genetic drift that has occurred on the branch. The scale bar shows ten times the average standard error of the entries in the sample covariance matrix.
(TIF)

S3 Fig Parsimony tree permutation length test showing a frequency distribution of tree lengths of maximum parsimony trees from 1000 artificially recombined datasets compared to the observed tree length (arrow) calculated for (a) all isolates partitioned into 2 species *B*. *dermatitidis* and *B*. *gilchristii*, (b) all isolates partitioned into 8 populations identified by STRUCTURE, (c) *B*. *dermatitidis* isolates partitioned into 4 populations as identified by STRUCTURE, and (d) *B*. *gilchristii* isolates partitioned into 4 populations as identified by STRUCTURE. Complete (black) and clone-corrected (gray) datasets are shown.
(TIF)

S1 Table Microsatellite fragment sizes detected in strains of *Blastomyces* spp. (DOCX)

S2 Table Characteristics of *Blastomyces dermatitidis* and *Blastomyces gilchristii* isolates studied. (DOCX)
