# Supplementary figures and images for: Phylogeographic Analysis of Blastomyces dermatitidis and Blastomyces gilchristii Reveals an Association with North American Freshwater Drainage Basins
Source: PLoS One. 2016 Jul 18;11(7):e0159396. doi: 10.1371/journal.pone.0159396 (PMC4948877; doi:10.1371/journal.pone.0159396)

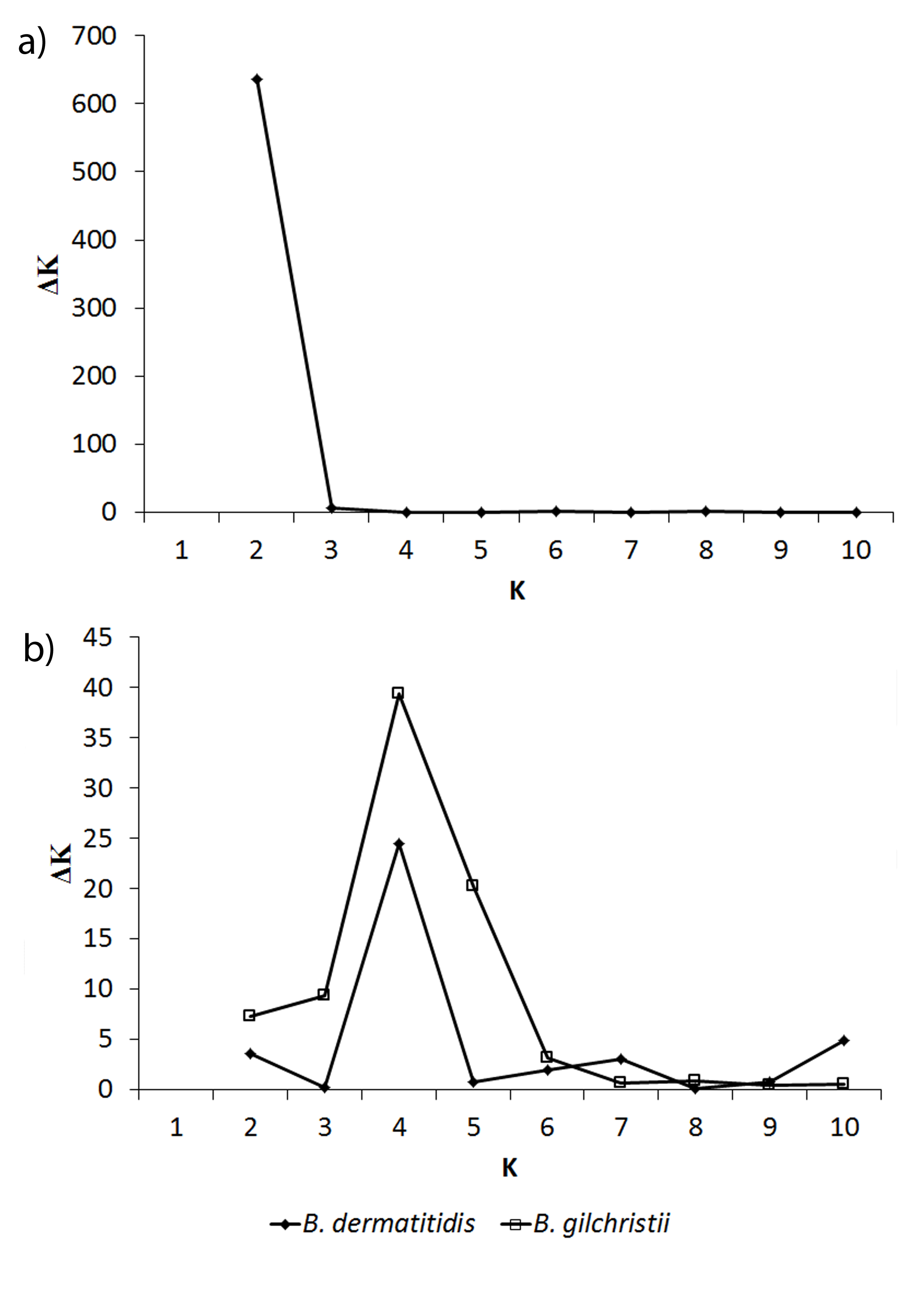

Supplement: S1 Fig — Where Delta K (ΔK) is equal to, ΔK = m(|L”(K)|)/s[L(K)]. (TIF) [file pone.0159396.s001.tif]

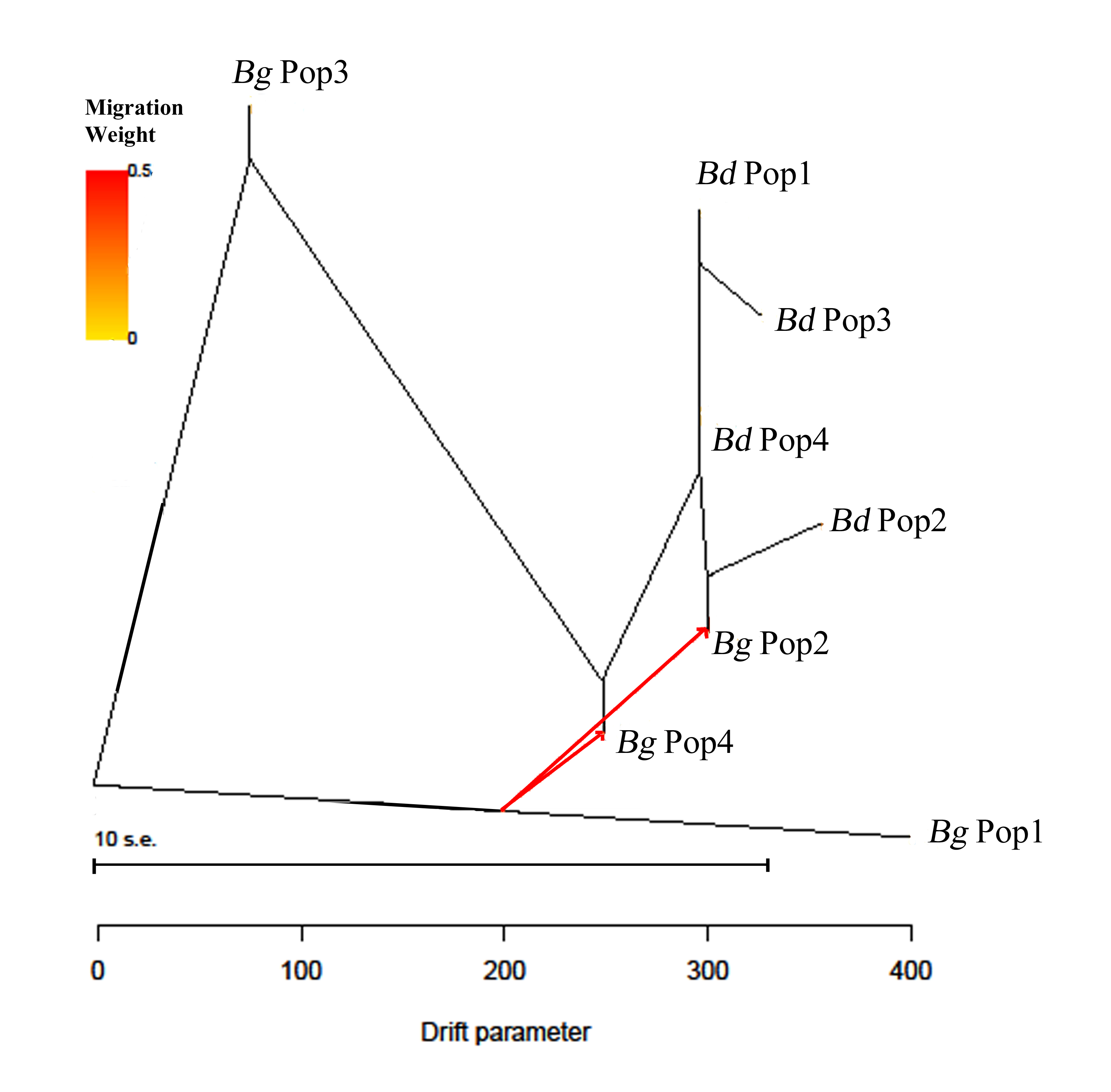

Supplement: S2 Fig — Migration arrows are coloured according to their weight. Horizontal branch lengths are proportional to the amount of genetic drift that has occurred on the branch. The scale bar shows ten times the average standard error of the entries in the sample covariance matrix. (TIF) [file pone.0159396.s002.tif]

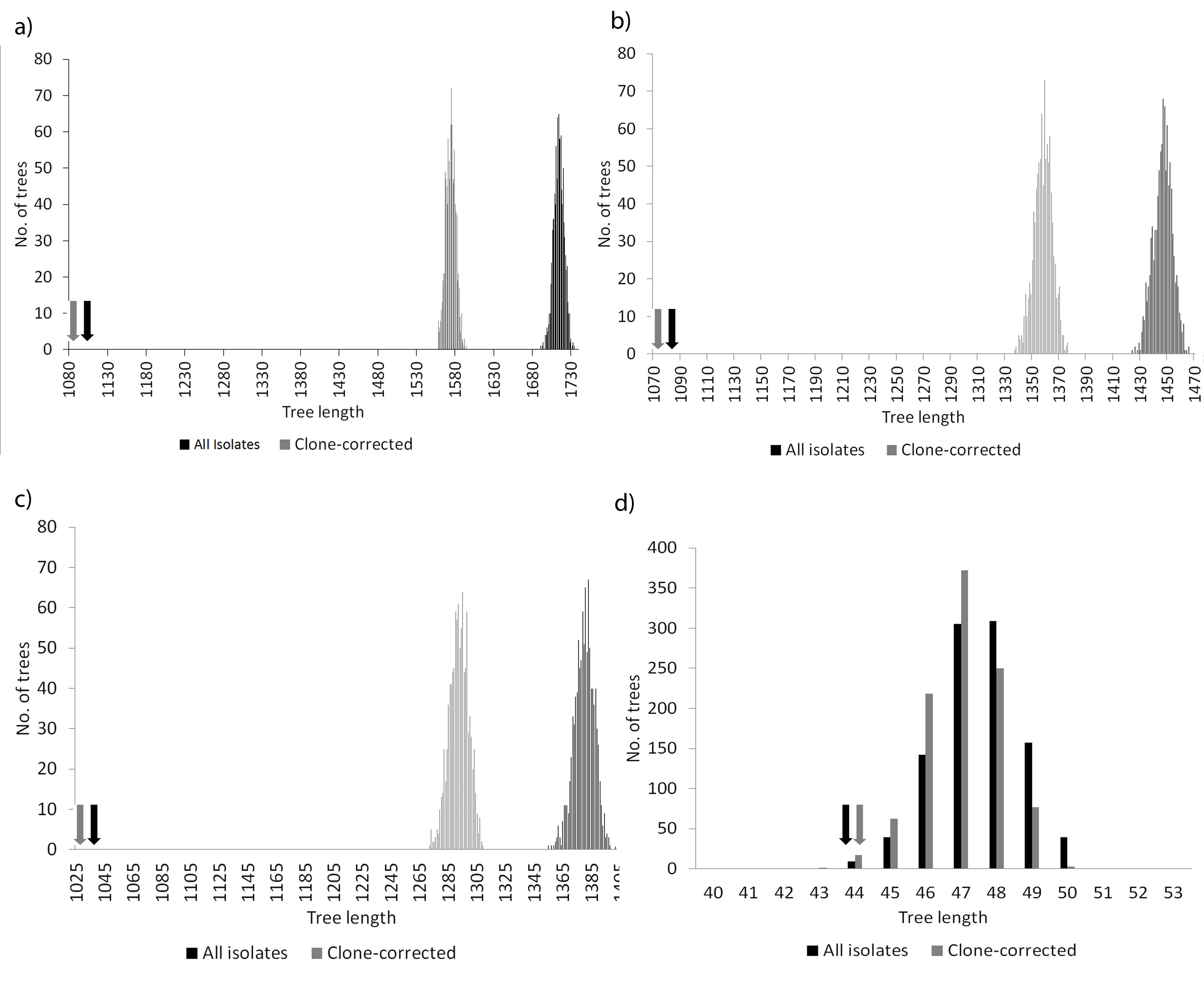

Supplement: S3 Fig — Complete (black) and clone-corrected (gray) datasets are shown. (TIF) [file pone.0159396.s003.tif]
