# Supplementary material for: Phylogeographic Analysis of Blastomyces dermatitidis and Blastomyces gilchristii Reveals an Association with North American Freshwater Drainage Basins
Source: PLoS One. 2016 Jul 18;11(7):e0159396. doi: 10.1371/journal.pone.0159396 (PMC4948877; doi:10.1371/journal.pone.0159396)
Supplement: S1 Table — (DOCX) [file pone.0159396.s004.docx]

**S1 Table: Microsatellite fragment sizes detected in strains of *Blastomyces***

|  | Microsatellite Size (bp) | | | | | | | | | | | | | | | | | | | | | | | | |
| --- | --- | --- | --- | --- | --- | --- | --- | --- | --- | --- | --- | --- | --- | --- | --- | --- | --- | --- | --- | --- | --- | --- | --- | --- | --- |
| Strain | 1 | 2 | 3 | 4 | 5 | 6 | 7 | 8 | 9 | 10 | 11 | 12 | 13 | 14 | 15 | 16 | 18 | 19 | 20 | 22 | 23 | 24 | 25 | 26 | 27 |
| ATCC 28306 | 247 | 203 | 218 | 229 | 214 | 261 | 258 | 209 | 216 | 211 | 185 | 251 | 203 | 264 | 201 | 254 | 213 | 240 | 233 | 190 | 222 | 169 | 251 | 167 | 219 |
| F2010035264 | 249 | 203 | 220 | 215 | 214 | 263 | 248 | 211 | 200 | 211 | 174 | 248 | 183 | 252 | 199 | 256 | 221 | 252 | 248 | 190 | 220 | 169 | 253 | 171 | 230 |
| F2011012490 | 247 | 210 | 210 | 231 | 222 | 263 | 248 | 211 | 198 | 223 | 173 | 272 | 183 | 252 | 201 | 264 | 222 | n/a | 227 | 190 | 220 | 169 | 249 | 171 | 231 |
| F2012014794 | 247 | 203 | 220 | 231 | 214 | 277 | 259 | 213 | 198 | 223 | 187 | n/a | 203 | 272 | 201 | 254 | 219 | 240 | 246 | 204 | 220 | 169 | 253 | 171 | 230 |
| F2012031974 | 247 | 205 | 199 | 227 | 214 | 235 | 259 | 213 | 198 | 223 | 172 | 251 | 183 | 252 | 203 | 260 | 205 | 238 | 248 | 190 | 220 | 169 | 253 | 184 | 231 |
| F2012034121 | 245 | 194 | 217 | 215 | 214 | 257 | 259 | 211 | 198 | 223 | 174 | 247 | 183 | 262 | 199 | 256 | 199 | 238 | 248 | 190 | 220 | 171 | 253 | 167 | 237 |
| F2013000710 | 247 | 203 | 220 | 227 | 214 | 259 | 248 | 211 | 198 | 211 | 184 | 255 | 183 | 252 | 199 | 234 | 219 | 238 | 248 | 190 | 220 | 169 | 251 | 171 | 229 |
| M06MY001520 | 247 | 203 | 220 | 231 | 222 | 263 | 248 | 213 | 198 | 211 | 175 | 249 | 183 | 252 | 201 | 262 | 221 | 230 | 233 | 192 | 217 | 169 | 252 | 167 | 230 |
| M07MY006203 | 247 | 203 | 220 | 231 | n/a^1^ | 261 | 248 | 213 | 198 | 211 | 190 | 249 | 203 | 268 | 201 | 256 | 221 | 230 | 233 | 192 | 217 | 169 | 252 | 171 | 230 |
| M07MY006625 | 247 | 203 | 220 | 231 | 222 | 261 | 248 | 213 | 198 | 211 | 175 | 249 | 183 | 268 | 201 | 262 | 221 | 230 | 233 | 190 | 217 | 169 | 252 | 171 | 225 |
| M08MY004008 | 247 | 210 | 218 | 231 | 222 | 261 | 248 | 211 | 198 | 211 | 185 | 249 | 183 | 252 | 199 | 254 | 221 | 230 | 233 | 192 | 217 | 169 | 248 | 198 | 235 |
| M10MY006786 | 247 | 203 | 226 | 227 | 214 | 275 | 248 | 211 | 198 | 213 | 174 | 247 | 183 | 252 | 201 | 256 | 199 | 242 | 243 | 192 | 220 | 169 | 244 | 171 | 225 |
| M11MY004917 | 247 | 203 | 220 | 231 | n/a | 261 | 248 | 213 | 198 | 211 | 175 | 249 | 183 | 252 | 201 | 254 | 221 | 230 | 233 | 192 | 217 | 169 | 252 | 198 | 225 |
| TB00011/2006 | 247 | 205 | 220 | 215 | 214 | 257 | 277 | 213 | 198 | 223 | 173 | 245 | 203 | 268 | 203 | 256 | 219 | 222 | 250 | 190 | 224 | 169 | 255 | 171 | 229 |
| TB00014/2008 | 245 | 205 | 200 | 227 | 214 | 261 | 259 | 209 | 198 | 211 | 185 | 241 | 183 | 252 | 203 | 256 | 219 | 222 | 248 | 190 | 220 | 169 | 253 | 171 | 230 |
| TB00042/2005 | 245 | 205 | 200 | 227 | 214 | 261 | 259 | 209 | 198 | 211 | 185 | 248 | 183 | 252 | 203 | 256 | 219 | 222 | 248 | 190 | 220 | 169 | 253 | 171 | 230 |
| UAMH 4042 | 245 | 203 | 226 | 231 | 214 | 263 | 248 | 209 | 198 | 211 | 175 | 249 | 183 | 252 | 201 | 254 | 199 | 238 | 248 | 192 | 217 | 169 | 254 | 167 | 233 |
| UAMH 5634 | 247 | 203 | 220 | 231 | 214 | 261 | 248 | 213 | 198 | 211 | 175 | 249 | 183 | 252 | 201 | 256 | 221 | 230 | 233 | 210 | 217 | 169 | 252 | 167 | 225 |
| UAMH 5635 | 247 | 203 | 220 | 231 | n/a | 261 | 248 | 213 | 198 | 211 | 175 | 249 | 183 | 252 | 201 | 256 | 221 | 230 | 233 | 210 | 217 | 169 | 252 | 167 | 225 |
| UAMH 7800 | 247 | 205 | 220 | 215 | 214 | 257 | 259 | 213 | 198 | 223 | 185 | 245 | 203 | 270 | 203 | n/a | 219 | 222 | 250 | 190 | 224 | 169 | 255 | 171 | 229 |
| 11PHO947 | 245 | 210 | 218 | 235 | 233 | 253 | 259 | 209 | 198 | 221 | 183 | 251 | 183 | 266 | 204 | 252 | 219 | 239 | 233 | 199 | 222 | 151 | 259 | 171 | 217 |
| 11PHO646 | 245 | 210 | 203 | 229 | 233 | 253 | 259 | 209 | 192 | 221 | 177 | 251 | 183 | 268 | 203 | 252 | 269 | 239 | 237 | 198 | 226 | 154 | 256 | 171 | 217 |
| 11PHO547 | 247 | 214 | 218 | 229 | 226 | 263 | 260 | 209 | 198 | 221 | 183 | 249 | 197 | 270 | 201 | 260 | 221 | 238 | 233 | 199 | 217 | 167 | 256 | 167 | 214 |
| 12PHO296 | 247 | 210 | 218 | 229 | 215 | 263 | 267 | 209 | 194 | 223 | 177 | 247 | 197 | 266 | 201 | 248 | 215 | 238 | 235 | 211 | 220 | 177 | 256 | 171 | 212 |
| 12PHO094 | 247 | 210 | 218 | 229 | n/a | 263 | 260 | 209 | 196 | 221 | 183 | 249 | 197 | 266 | 201 | 250 | 236 | 232 | 250 | 206 | 222 | 167 | 250 | 171 | 214 |
| 12PHO950 | 247 | 214 | 195 | 229 | 234 | 263 | 260 | 209 | 194 | 221 | 183 | 249 | 212 | 266 | 203 | 250 | 213 | 232 | 252 | 208 | 217 | 167 | 256 | 188 | 214 |
| 13PHO345 | 245 | 202 | 216 | 237 | 208 | 253 | 259 | 226 | 192 | 221 | 177 | 241 | 183 | 266 | 201 | 248 | 259 | 242 | 233 | 198 | 217 | 167 | 252 | 171 | 212 |
| 13PHO534 | 247 | 210 | 222 | 229 | 215 | 255 | 267 | 209 | 194 | 221 | 177 | 247 | 197 | 270 | 201 | 262 | 248 | 238 | 252 | 199 | 222 | 175 | 248 | 188 | 237 |
| 13PHO421 | 247 | 210 | 224 | 229 | 214 | 253 | 267 | 209 | 198 | 221 | 183 | 247 | 197 | 266 | 201 | 250 | 236 | 238 | 231 | 206 | 222 | 167 | 250 | 171 | 214 |
| DI 13-69 | 247 | 208 | 216 | 231 | 233 | 253 | 260 | 209 | 194 | 213 | 182 | 249 | 183 | 266 | 203 | 240 | 227 | 246 | 233 | 208 | 217 | 177 | 254 | 169 | 212 |
| DI 13-71 | 245 | 203 | 224 | 235 | 233 | 253 | 260 | 209 | 192 | 219 | 182 | 241 | 183 | 264 | 203 | 260 | 261 | 239 | 235 | 208 | 217 | 165 | 248 | 169 | 214 |
| DI 13-73 | 247 | 208 | 216 | 231 | 233 | 253 | 260 | 209 | 194 | 213 | 182 | 249 | 183 | 266 | 203 | 240 | 227 | 246 | 233 | 208 | 217 | 177 | 254 | 169 | 212 |
| DI 13-74 | 245 | 210 | 218 | 235 | 233 | 265 | 261 | 226 | 194 | 213 | 183 | 241 | 183 | 266 | 203 | 256 | 259 | 239 | 237 | 208 | 217 | 154 | 250 | 169 | 212 |
| DI 13-78 | 245 | 203 | 224 | 235 | 233 | 253 | 260 | 209 | 192 | 219 | 182 | 243 | 183 | 264 | 203 | 260 | 261 | 239 | 235 | 208 | 217 | 165 | 248 | 169 | 214 |
| LSPQ-00666 | 245 | 202 | 210 | 217 | 200 | 296 | 273 | 214 | 190 | n/a | 154 | 243 | 191 | 262 | 199 | 250 | 227 | 226 | 231 | 191 | 219 | 167 | 248 | 167 | 227 |
| LSPQ-00755 | 245 | 208 | 224 | 235 | 233 | 261 | 259 | 224 | 192 | 221 | 188 | 243 | 183 | 266 | 203 | 252 | 230 | 232 | 235 | 198 | 217 | 154 | 248 | 169 | 209 |
| LSPQ-00854 | 245 | 203 | 224 | 235 | 214 | 253 | 260 | 209 | 198 | 221 | 182 | 243 | 183 | 264 | 203 | 258 | 261 | 232 | 233 | 204 | 217 | 154 | 248 | 173 | 214 |
| LSPQ-00859 | 245 | 208 | 224 | 237 | 233 | 253 | 260 | 226 | 192 | 211 | 181 | 243 | 183 | 264 | 203 | 258 | 219 | 240 | 233 | 206 | 205 | 165 | 248 | 173 | 214 |
| LSPQ-00876 | 245 | 210 | 218 | 235 | 239 | 251 | 260 | 226 | 194 | 213 | 182 | 256 | 183 | 264 | 203 | 256 | 259 | 239 | 237 | 208 | 217 | 154 | 248 | 169 | 212 |
| LSPQ-00884 | 247 | 208 | 224 | 231 | 233 | 265 | 264 | 226 | 198 | 221 | 183 | 243 | 183 | 266 | 203 | 250 | 219 | 239 | 231 | 198 | 222 | 154 | 256 | 169 | n/a |
| LSPQ-00941 | 247 | 210 | 201 | 231 | 233 | 265 | 262 | 209 | 192 | 221 | 177 | 247 | 183 | 270 | 203 | 252 | 269 | 239 | 233 | 198 | 217 | 167 | 256 | 169 | 214 |
| LSPQ-00962 | 247 | 210 | 224 | 231 | 214 | 247 | 260 | 209 | 192 | 221 | 192 | 243 | 183 | 266 | 203 | 252 | 219 | 232 | 235 | 198 | 217 | 175 | 256 | 171 | 212 |
| LSPQ-01031 | 245 | 208 | 224 | 235 | 233 | 253 | 260 | 209 | 198 | 221 | 181 | 243 | 183 | 264 | 203 | 250 | 221 | 240 | 233 | 204 | 205 | 154 | 248 | 173 | 214 |
| M1954 | 247 | 208 | 216 | 235 | 233 | 277 | 260 | 213 | 194 | 221 | 181 | 243 | 199 | 266 | 201 | 252 | 256 | 239 | 248 | 206 | 217 | 165 | 254 | 169 | 212 |
| M2166 | 245 | 202 | 210 | 217 | 200 | 296 | 273 | 214 | 190 | n/a | 154 | 243 | 191 | 262 | 199 | 250 | 227 | 226 | 231 | 191 | 219 | 167 | 248 | 167 | 227 |
| M2574 | 245 | 202 | 210 | 217 | 200 | 296 | 276 | 214 | 190 | n/a | 154 | 243 | 191 | 262 | 199 | 250 | 227 | 226 | 231 | 191 | 219 | 167 | 248 | 167 | 227 |
| M3626 | 247 | 208 | 216 | 235 | 233 | 277 | 271 | 213 | 194 | 221 | 181 | 241 | 183 | 266 | 201 | 252 | 256 | 239 | 248 | 206 | 217 | 165 | 254 | 169 | 212 |
| MYCO-01451 | 245 | 210 | 216 | 235 | 233 | 265 | 259 | 209 | 200 | 221 | 182 | 251 | 195 | 266 | 199 | 250 | 256 | 232 | 233 | 199 | 222 | 154 | 248 | 169 | 212 |
| SF01520/2010 | 247 | 210 | 218 | 227 | 237 | 253 | 260 | 209 | 194 | 221 | 189 | 251 | 195 | 266 | 201 | 254 | 246 | 238 | 250 | 199 | 222 | 167 | 252 | 169 | 237 |
| SF06587/2009 | 247 | 210 | 218 | 229 | 214 | 255 | 267 | 211 | 198 | 221 | 183 | 247 | 195 | 266 | 199 | 250 | 213 | 232 | 233 | 199 | 217 | 167 | 256 | 188 | 209 |
| SF08339/2009 | 247 | 210 | 216 | 229 | 214 | 253 | 267 | 209 | 194 | 223 | 177 | 247 | 193 | 266 | 201 | 248 | 244 | 238 | 233 | 199 | 205 | 154 | 248 | 167 | 214 |
| SF12545/2009 | 247 | 214 | 218 | 229 | 226 | 263 | 260 | 209 | 198 | 221 | 183 | 249 | 197 | 270 | 201 | 260 | 221 | 238 | 233 | 199 | 217 | 167 | 256 | 167 | 214 |
| 371 | 247 | 212 | 222 | 225 | 216 | 251 | 261 | 209 | 196 | 221 | 185 | 255 | 197 | 264 | 204 | 258 | 234 | 242 | 246 | 202 | 220 | 167 | 250 | 191 | 209 |
| 663 | 247 | 212 | 222 | 225 | 200 | 259 | 258 | 213 | 204 | 219 | 185 | 247 | 197 | 264 | 199 | 254 | 221 | 244 | 246 | 202 | 220 | 171 | 250 | 186 | 214 |
| 664 | 247 | 210 | 212 | 225 | 218 | 259 | 261 | 209 | 204 | 223 | 185 | 247 | 197 | 264 | 206 | 256 | 227 | 244 | 244 | 191 | 205 | 169 | 255 | 171 | 214 |
| ATCC 26197 (GA-1) | 247 | 212 | 222 | 225 | 218 | 251 | 261 | 211 | 196 | 221 | 185 | 251 | 197 | 264 | 199 | 248 | 263 | 244 | 244 | 204 | 205 | 165 | 251 | 190 | 209 |
| ATCC 60916 (A2) | 247 | 212 | 220 | 225 | 214 | 259 | 258 | 213 | 196 | 219 | 185 | 247 | 197 | 264 | 206 | 254 | 221 | 244 | 246 | 202 | 220 | 171 | 255 | 186 | 233 |
| DI 13-61 | 247 | 213 | 212 | 229 | 233 | 251 | 258 | 209 | 198 | 219 | 183 | 255 | 183 | 264 | 199 | 254 | 265 | 234 | 233 | 202 | 220 | 171 | 251 | 186 | 209 |
| Gu | 247 | 212 | 220 | 225 | 214 | 259 | 258 | 213 | 196 | 219 | 185 | 247 | 197 | 264 | 206 | 254 | 221 | 244 | 246 | 202 | 220 | 171 | 255 | 186 | 233 |
| K966 | 247 | 212 | 222 | 225 | 218 | 259 | 260 | 209 | 196 | 221 | 185 | 250 | 197 | 264 | 199 | 256 | 234 | 242 | 246 | 202 | 205 | 165 | 251 | 171 | 233 |
| Ro | 247 | 202 | 201 | 225 | 226 | 247 | 260 | 232 | 198 | 217 | 167 | 249 | 185 | 266 | 203 | 256 | 225 | 238 | 234 | 210 | 219 | 171 | 252 | 183 | 220 |
| UAB 00001 | 247 | 210 | 216 | 225 | 218 | 251 | 261 | 209 | 196 | 221 | 183 | 251 | 197 | 264 | 199 | 258 | 211 | 242 | 244 | 198 | 220 | 171 | 255 | 187 | 235 |
| UAB 00018 | 247 | 212 | 222 | 225 | 226 | 247 | 260 | 214 | 194 | 221 | 167 | 249 | 185 | 266 | 203 | 256 | 219 | 242 | 244 | 198 | 219 | 171 | 252 | 183 | 220 |
| UAB 00019 | 245 | 202 | 202 | 225 | 218 | 251 | 261 | 214 | 194 | 221 | 172 | 249 | 185 | 266 | 203 | 258 | 225 | 238 | 234 | 210 | 219 | 171 | 252 | 183 | 220 |
| UAB 00024 | 247 | 202 | 204 | 225 | 226 | 247 | 260 | 209 | 204 | 221 | 183 | 249 | 197 | 264 | 203 | 256 | 219 | 242 | 244 | 210 | 216 | 172 | 255 | 190 | 209 |
| UAB 00025 | 247 | 212 | 220 | 225 | 218 | 251 | 261 | 209 | 196 | 221 | 185 | 255 | 197 | 264 | 199 | 256 | 219 | 242 | 244 | 208 | 217 | 165 | 250 | 191 | 233 |
| UAB 00026 | 232 | 210 | 216 | n/a | 226 | 247 | 260 | 209 | 194 | 219 | 181 | 247 | 185 | 266 | 203 | 256 | 220 | 244 | 237 | 210 | 217 | 172 | 254 | 183 | 220 |
| UAB 00033 | 247 | 210 | 216 | 225 | 218 | 285 | 261 | 209 | 204 | 221 | 183 | 251 | 197 | 264 | 204 | 248 | 219 | 242 | 244 | 202 | 205 | 165 | 251 | 190 | 209 |
| UAB 00034 | 247 | 202 | 202 | 225 | 218 | 251 | 261 | 209 | 204 | 221 | 185 | 247 | 197 | 264 | 199 | 256 | 219 | 242 | 244 | 202 | 217 | 165 | 255 | 190 | 209 |
| UAB 00036 | 247 | 210 | 216 | 225 | 226 | 247 | 260 | 209 | 194 | 219 | 181 | 247 | 185 | 266 | 203 | 256 | 220 | 244 | 237 | 210 | 216 | 172 | 254 | 183 | 220 |
| UAB 00037 | 247 | 212 | 222 | 225 | 218 | 285 | 261 | 209 | 204 | 219 | 185 | 255 | 197 | 264 | 199 | 258 | 219 | 242 | 244 | 202 | 220 | 171 | 250 | 186 | 209 |
| UAB 00038 | 247 | 212 | 216 | 225 | 218 | 285 | 258 | 209 | 196 | 219 | 183 | 255 | 197 | 264 | 199 | 258 | 219 | 244 | 244 | 208 | 220 | 171 | 255 | 186 | 209 |
| UAB 00040 | 249 | 202 | 201 | 225 | 218 | 251 | 261 | 209 | 204 | 221 | 172 | 249 | 185 | 266 | 203 | 256 | 225 | 244 | 234 | 210 | 219 | 171 | 252 | 183 | 220 |
| UAB 00041 | 245 | 202 | 202 | 225 | 218 | 251 | 261 | 209 | 204 | 221 | 172 | 249 | 185 | 266 | 203 | 256 | 225 | 244 | 234 | 210 | 219 | 171 | 252 | 183 | 220 |
| UAB 00043 | 247 | 212 | 222 | 225 | 218 | 285 | 258 | 214 | 194 | 221 | 183 | 251 | 183 | 264 | 203 | 258 | 225 | 242 | 244 | 198 | 219 | 171 | 250 | 183 | 220 |
| UAB 00047 | 247 | 210 | 216 | 225 | 218 | 285 | 261 | 209 | 196 | 219 | 183 | 251 | 197 | 264 | 204 | 248 | 219 | 244 | 244 | 202 | 205 | 165 | 255 | 190 | 214 |
| 397 | 246 | 203 | 212 | 227 | 220 | 263 | 259 | 209 | 194 | 223 | 186 | 247 | 183 | 264 | 199 | 244 | 201 | 240 | 235 | 206 | 220 | 169 | 253 | 181 | 237 |
| 12MYC01064 | 247 | 214 | 197 | 215 | 226 | 263 | 246 | 209 | 196 | 225 | 187 | 255 | 183 | 276 | 201 | 262 | 199 | 238 | 233 | 202 | 218 | 167 | 254 | 167 | 232 |
| 12MYC0354 | 247 | 214 | 197 | 215 | 226 | 263 | 246 | 209 | 196 | 225 | 187 | 255 | 183 | 276 | 201 | 262 | 199 | 238 | 233 | 202 | 218 | 167 | 254 | 167 | 232 |
| 12MYC0592 | 246 | 214 | 197 | 215 | 226 | 263 | 246 | 209 | 204 | 215 | 174 | 255 | 183 | 270 | 201 | 262 | 219 | 236 | 226 | n/a | 220 | 167 | 254 | 169 | 237 |
| 12MYC0593 | 246 | 214 | 197 | 225 | 216 | 251 | 260 | 209 | 204 | 215 | 174 | 251 | 183 | 272 | 201 | 256 | 219 | 236 | 226 | n/a | 220 | 167 | 254 | 167 | 235 |
| 13MYC0115 | 246 | 214 | 197 | 215 | 226 | 263 | 260 | 209 | 196 | 225 | 187 | 255 | 183 | 270 | 201 | 262 | 217 | 238 | 226 | n/a | 220 | 167 | 254 | 167 | 232 |
| ATCC MYA-2586 (ER-3) | 249 | 210 | 216 | 215 | 214 | 273 | 261 | 209 | 194 | 213 | 189 | 243 | 203 | 262 | 204 | 256 | 199 | 238 | 235 | 212 | 217 | 167 | 253 | 171 | 237 |
| CBS 673.68 (ATCC 18187) | 245 | 210 | 231 | 229 | 214 | 263 | 258 | 209 | 216 | 221 | 173 | 245 | 199 | 264 | 204 | 256 | 219 | 242 | 235 | 206 | 224 | 159 | 251 | 167 | 237 |
| CBS 674.68 (ATCC 18188) | 246 | 210 | 231 | 227 | 208 | 251 | 260 | 209 | 216 | 221 | 172 | 255 | 197 | 264 | 204 | 258 | 219 | 242 | 235 | 206 | 224 | 159 | 251 | 167 | 237 |
| CH-10 | 245 | 213 | 207 | 227 | 224 | 265 | 260 | 213 | 214 | 221 | 181 | 243 | 183 | 268 | 201 | 250 | 217 | 244 | 234 | 188 | 224 | 173 | 234 | 188 | 233 |
| CH-6 | 245 | 213 | 207 | 227 | 224 | 265 | 260 | 213 | 214 | 221 | 181 | 243 | 183 | 268 | 201 | 250 | 217 | 244 | 234 | 188 | 224 | 173 | 234 | 188 | 233 |
| DI 13-101 | 251 | 210 | 231 | 229 | 214 | 263 | 260 | 207 | 194 | 221 | 165 | 243 | 183 | 252 | 201 | 240 | 209 | 238 | 233 | 206 | 217 | 171 | 253 | 167 | 219 |
| DI 13-12 | 245 | 210 | 217 | 227 | 224 | 268 | 260 | 209 | 190 | 211 | 172 | 243 | 207 | 272 | 201 | 258 | 217 | 242 | 231 | 206 | 220 | 159 | 249 | 167 | 237 |
| DI 13-15 | 247 | 214 | 221 | 235 | 214 | 265 | 264 | 209 | 194 | 205 | 173 | 249 | 207 | 262 | 203 | 256 | 219 | 240 | 248 | 204 | 220 | 169 | 249 | 167 | 237 |
| DI 13-18 | 245 | 210 | 218 | 215 | 208 | 268 | 260 | 218 | 194 | 221 | 183 | 247 | 199 | 266 | 204 | 262 | 221 | 240 | 235 | 212 | 220 | 167 | 256 | 181 | 227 |
| DI 13-20 | 246 | 210 | 207 | 227 | 226 | 275 | 260 | n/a | 204 | 213 | 186 | 247 | 205 | 264 | 201 | 256 | 221 | 234 | 237 | n/a | 217 | 167 | 234 | 184 | 239 |
| DI 13-21 | 245 | 202 | 201 | 231 | 214 | 261 | 260 | 209 | 198 | 213 | 174 | 234 | 197 | 274 | 201 | 260 | 205 | 239 | 235 | 208 | 220 | 169 | 250 | 181 | 238 |
| DI 13-28 | 247 | 216 | 231 | 225 | 214 | 263 | 260 | 216 | 196 | 213 | 171 | 245 | 203 | 264 | 193 | 254 | 221 | 242 | 246 | 211 | 220 | 169 | 251 | 184 | 237 |
| DI 13-30 | 247 | 213 | 220 | 227 | 208 | 251 | 260 | 209 | 198 | 223 | 174 | 255 | 205 | 264 | 204 | 254 | 203 | 238 | 248 | 212 | 224 | 159 | 256 | 171 | 237 |
| DI 13-36 | 247 | n/a | 220 | 223 | 214 | 259 | 260 | 209 | 202 | 217 | 172 | 250 | 183 | 266 | 203 | 252 | 211 | 244 | 254 | 210 | 222 | 161 | 256 | 190 | 235 |
| DI 13-37 | 247 | n/a | 220 | 223 | 214 | 259 | 260 | 209 | 184 | 217 | 172 | 250 | 183 | 266 | 203 | 252 | 211 | 244 | 254 | 210 | 221 | 161 | 256 | 190 | 235 |
| DI 13-57 | 245 | 202 | 231 | 229 | n/a | 269 | 260 | 213 | 201 | 215 | 178 | 249 | 192 | 252 | 203 | 282 | 211 | n/a | 235 | 208 | 220 | 159 | 254 | 188 | 209 |
| DI 13-58 | 247 | 203 | 203 | 233 | n/a | 247 | 260 | 207 | 201 | 215 | 167 | 241 | 183 | 272 | 203 | 260 | 213 | 244 | 241 | 208 | 220 | 159 | 248 | 183 | 225 |
| DI 13-60 | 245 | 208 | 203 | 229 | 216 | 247 | 259 | 209 | 204 | 215 | 165 | 245 | 183 | 266 | 203 | 250 | 211 | 246 | 231 | 210 | 222 | 159 | 234 | 175 | 236 |
| DI 13-63 | 247 | 210 | 216 | 227 | n/a | 251 | 260 | 209 | 190 | 211 | 195 | 256 | 207 | 272 | 201 | 258 | 234 | 238 | 235 | 204 | 220 | 159 | 252 | 167 | 237 |
| DI 13-64 | 247 | 203 | 193 | 215 | 220 | 245 | 260 | 209 | 201 | 238 | 171 | 247 | 183 | n/a | 199 | 236 | 244 | 240 | 252 | 206 | 219 | 167 | 252 | 175 | 233 |
| DI 13-66 | 247 | 203 | n/a | 215 | 226 | 269 | 260 | 230 | 202 | 217 | 183 | 247 | 183 | 266 | 199 | 242 | 213 | 246 | 239 | 210 | 222 | 171 | 252 | 175 | 233 |
| DI 13-67 | 245 | 213 | 216 | 229 | 222 | 269 | 260 | 209 | 201 | 221 | 183 | 251 | 183 | 264 | 197 | 254 | 211 | 240 | 231 | 214 | 224 | 173 | 235 | 175 | 230 |
| DI 13-80 | 246 | 210 | 212 | 227 | 214 | 263 | 260 | 218 | 196 | 217 | 185 | 251 | 183 | 264 | 201 | 264 | 209 | 238 | 233 | 206 | 220 | 161 | 251 | 167 | 220 |
| DI 13-81 | 247 | 210 | 201 | 231 | 222 | 263 | 262 | 209 | 198 | 213 | 165 | 243 | 197 | 274 | 204 | 246 | 227 | 242 | 226 | 194 | 220 | 169 | 256 | 181 | 237 |
| DI 13-83 | 251 | 210 | 207 | 227 | 224 | 263 | 258 | 216 | 194 | 223 | 175 | 245 | 183 | 260 | 201 | 254 | 213 | 236 | 244 | 206 | 220 | 169 | 252 | 204 | 237 |
| DI 13-86 | 251 | 210 | 207 | 227 | 224 | 263 | 258 | 216 | 194 | 223 | 175 | 245 | 183 | 260 | 201 | 254 | 213 | 236 | 244 | 206 | 220 | 169 | 252 | 204 | 237 |
| DI 13-9 | 245 | 210 | 214 | 213 | 222 | 269 | 260 | 209 | 198 | 221 | 184 | 247 | 203 | 266 | 204 | 258 | 221 | 240 | 235 | 212 | 220 | 167 | 256 | 181 | 235 |
| DI 13-96 | 247 | 208 | 201 | 215 | 222 | 273 | 260 | 214 | 202 | 217 | 177 | 247 | n/a | 266 | 199 | 242 | 211 | 246 | 231 | 198 | 220 | 159 | 248 | 175 | 235 |
| En | 246 | 203 | 201 | 235 | 214 | 263 | 259 | 211 | 204 | 223 | 171 | 247 | 183 | 264 | 204 | 256 | 213 | 232 | 235 | 213 | 220 | 159 | 253 | 167 | 234 |
| F2011027401 | 246 | 204 | 216 | 215 | 214 | 263 | 261 | 211 | 196 | 221 | 173 | 243 | 203 | 262 | 204 | 256 | 199 | 238 | 237 | 212 | 220 | 167 | 247 | 198 | 237 |
| MICH-1 | 247 | 210 | 216 | 227 | n/a | 251 | 260 | 209 | 190 | 211 | 195 | 253 | 207 | 272 | 201 | 258 | 234 | 238 | 235 | 204 | 220 | 159 | 252 | 167 | 237 |
| MICH-2 | 247 | 214 | 218 | 237 | 208 | 245 | 260 | 209 | 194 | 221 | 182 | 247 | 183 | 266 | 204 | 248 | 217 | 241 | 227 | 206 | 220 | 167 | 253 | 167 | n/a |
| MICH-3 | 246 | 214 | 197 | 215 | 224 | 265 | 262 | 209 | 204 | 215 | 174 | 255 | 183 | 270 | 201 | 262 | 217 | 238 | 226 | n/a | 220 | 167 | 244 | 167 | 237 |
| SACR | 245 | 208 | 204 | 229 | 216 | 269 | 259 | 209 | 204 | 217 | 169 | 245 | 183 | 266 | 193 | 258 | 199 | 240 | 251 | 210 | 222 | 171 | 234 | 175 | 236 |
| SF06266/2009 | 246 | 204 | 216 | n/a | 214 | 263 | 261 | 209 | 194 | 221 | 173 | 243 | 203 | 262 | 204 | n/a | 199 | n/a | 237 | 212 | 221 | 167 | 247 | 198 | 237 |
| SU-SACS | 245 | 208 | 203 | 229 | 216 | 269 | 259 | 209 | 204 | 217 | 169 | 245 | 183 | 266 | 193 | 258 | 199 | 240 | 251 | 210 | 222 | 171 | 234 | 175 | 236 |
| UAMH 5584 | 247 | 208 | 211 | 221 | 214 | 271 | 262 | 209 | 192 | 225 | 163 | 247 | 183 | 266 | 203 | 260 | 199 | 238 | 248 | 206 | 222 | 169 | 254 | 167 | 229 |
| 12PHO936 | 249 | 211 | 208 | 219 | 214 | 285 | 277 | 214 | 194 | 213 | 154 | 243 | 191 | 260 | 199 | 250 | 199 | 228 | 220 | 200 | 217 | 165 | 248 | 167 | 231 |
| 12PHO819 | 249 | 211 | 208 | 219 | 214 | 285 | 277 | 214 | 194 | 213 | 154 | 243 | 191 | 260 | 199 | 250 | 199 | 228 | 220 | 200 | 217 | 165 | 248 | 167 | 231 |
| 12PHO858 | 248 | 211 | 207 | 219 | 214 | 285 | 277 | 214 | 194 | 213 | 154 | 243 | 191 | 260 | 199 | 250 | 199 | 228 | 220 | 200 | 217 | 165 | 248 | 167 | 231 |
| 12PHO859 | 249 | 211 | 207 | 219 | 214 | 285 | 277 | 214 | 194 | 213 | 154 | 243 | 191 | 260 | 199 | 250 | 199 | 228 | 220 | 200 | 217 | 165 | 248 | 167 | 231 |
| F2011036969 | 249 | 211 | 207 | 219 | 214 | 285 | 277 | 214 | 194 | 213 | 154 | 243 | 191 | 260 | 199 | 252 | 219 | 228 | 220 | 200 | 217 | 165 | 248 | 167 | 231 |
| F2013001127 | 249 | 211 | 207 | 219 | 214 | 285 | 277 | 214 | 194 | 213 | 154 | 243 | 191 | 260 | 199 | 250 | 199 | 228 | 220 | 200 | 217 | 165 | 248 | 167 | 231 |
| M08MY007367 | 249 | 211 | 207 | 219 | 214 | 285 | 277 | 214 | 194 | 213 | 154 | 243 | 191 | 260 | 199 | 250 | 199 | 228 | 220 | 200 | 217 | 165 | 248 | 167 | 231 |
| M09MY002766 | 249 | 211 | 207 | 219 | 214 | 285 | 277 | 214 | 194 | 213 | 154 | 243 | 191 | 260 | 199 | 250 | 199 | 228 | 220 | 200 | 217 | 165 | 248 | 167 | 231 |
| M10MY005485 | 249 | 211 | 207 | 219 | 214 | 285 | 277 | 214 | 194 | 213 | 154 | 243 | 191 | 260 | 199 | 250 | 199 | 228 | 220 | 200 | 217 | 165 | 248 | 167 | 231 |
| M11MY006516 | 249 | 211 | 207 | 219 | 214 | 285 | 277 | 214 | 194 | 213 | 154 | 243 | 191 | 260 | 199 | 250 | 199 | 228 | 220 | 200 | 217 | 165 | 248 | 167 | 231 |
| TB00002/2008 | 249 | 211 | 208 | 219 | 214 | 285 | 277 | 214 | 194 | 213 | 154 | 243 | 191 | 260 | 199 | 250 | 199 | 228 | 220 | 200 | 217 | 165 | 248 | 167 | 231 |
| TB00016/2005 | 249 | 211 | 208 | 219 | 214 | 285 | 277 | 214 | 194 | 213 | 154 | 243 | 191 | 260 | 199 | 250 | 199 | 228 | 220 | 200 | 217 | 165 | 248 | 167 | 231 |
| TB00017/2006 | 249 | 211 | 208 | 219 | 214 | 285 | 277 | 214 | 194 | 213 | 154 | 243 | 191 | 260 | 199 | 250 | 199 | 228 | 220 | 200 | 217 | 165 | 248 | 167 | 231 |
| TB00018/2005 | 249 | 211 | 207 | 219 | 214 | 285 | 277 | 214 | 194 | 213 | 154 | 243 | 191 | 260 | 199 | 250 | 199 | 228 | 220 | 200 | 217 | 165 | 248 | 167 | 231 |
| TB00018/2006 | 249 | 211 | 207 | 219 | 214 | 285 | 277 | 214 | 194 | 213 | 154 | 243 | 191 | 260 | 199 | 250 | 199 | 228 | 220 | 200 | 217 | 165 | 248 | 167 | 231 |
| TB00019/2005 | 249 | 211 | 208 | 219 | 214 | 285 | 277 | 214 | 194 | 213 | 154 | 243 | 191 | 260 | 199 | 250 | 199 | 228 | 220 | 200 | 217 | 165 | 248 | 167 | 231 |
| TB00022/2006 | 249 | 211 | 207 | 219 | 214 | 285 | 277 | 214 | 194 | 213 | 154 | 243 | 191 | 260 | 199 | 250 | 199 | 228 | 220 | 200 | 217 | 165 | 248 | 167 | 231 |
| TB00023/2005 | 249 | 211 | 207 | 219 | 214 | 285 | 277 | 214 | 194 | 213 | 154 | 243 | 191 | 260 | 199 | 250 | 199 | 228 | 220 | 200 | 217 | 165 | 248 | 167 | 231 |
| TB00025/2005 | 249 | 211 | 208 | 219 | 214 | 285 | 277 | 214 | 194 | 213 | 154 | 243 | 191 | 260 | 199 | 250 | 199 | 228 | 220 | 200 | 217 | 165 | 248 | 167 | 231 |
| TB00029/2006 | 249 | 211 | 207 | 219 | 214 | 285 | 277 | 214 | 194 | 213 | 154 | 243 | 191 | 260 | 199 | 250 | 199 | 228 | 220 | 200 | 217 | 165 | 248 | 167 | 231 |
| TB00032/2006 | 249 | 211 | 208 | 219 | 214 | 285 | 277 | 214 | 194 | 213 | 154 | 243 | 191 | 260 | 199 | 250 | 199 | 228 | 220 | 200 | 217 | 167 | 248 | 167 | 231 |
| TB00037/2008 | 249 | 211 | 208 | 219 | 214 | 285 | 277 | 214 | 194 | 213 | 154 | 243 | 191 | 260 | 199 | 250 | 199 | 228 | 220 | 200 | 217 | 165 | 248 | 167 | 231 |
| TB00038/2005 | 249 | 211 | 207 | 219 | 214 | 285 | n/a | 214 | 194 | 213 | 154 | 243 | 191 | 260 | 199 | 250 | 199 | 228 | 220 | 200 | 217 | 165 | 248 | 167 | 231 |
| TB00040/2005 | 249 | 211 | 207 | 219 | 214 | 285 | 277 | 214 | 194 | 213 | 154 | 243 | 191 | 260 | 199 | 250 | 199 | 228 | 220 | 200 | 217 | 165 | 248 | 167 | 231 |
| F2010034629 | 246 | 203 | 205 | 219 | 214 | 283 | 262 | 214 | 196 | 213 | 154 | 243 | 191 | 260 | 199 | 252 | 199 | 238 | 220 | 200 | 217 | 165 | 248 | 167 | 231 |
| F2010037690 | 249 | 211 | 205 | 219 | 214 | 283 | 262 | 214 | 196 | 213 | 154 | 243 | 191 | 260 | 199 | 252 | 199 | 238 | 220 | 200 | 217 | 165 | 248 | 167 | 231 |
| F2011026044 | 249 | 211 | 205 | 219 | 214 | 283 | 262 | 214 | 196 | 213 | 154 | 243 | 191 | 260 | 199 | 252 | 199 | 238 | 220 | 200 | 217 | 165 | 248 | 167 | 231 |
| F252 | 249 | 211 | 205 | 219 | 214 | 283 | 262 | 214 | 196 | 213 | 154 | 243 | 191 | 260 | 199 | 252 | 199 | 238 | 220 | 200 | 217 | 165 | 248 | 167 | 231 |
| 10PHO703 | 247 | 211 | 207 | 217 | 214 | 341 | 277 | 214 | 194 | 213 | 154 | 253 | 193 | 264 | 199 | 252 | 199 | 228 | 220 | 200 | 217 | 165 | 248 | 167 | 231 |
| 10PHO673 | 247 | 198 | 208 | 217 | 214 | 273 | 279 | 214 | 194 | 213 | 154 | 253 | 193 | 264 | 199 | 250 | 203 | 228 | 222 | 204 | 217 | 165 | 248 | 179 | 231 |
| 11PHO185 | 247 | 211 | 208 | 217 | 214 | 275 | 277 | 214 | 194 | 213 | 154 | 251 | 193 | 264 | 199 | 252 | 199 | 228 | 220 | 200 | 217 | 165 | 248 | 167 | 231 |
| 12PHO241 | 247 | 211 | 208 | 217 | 214 | 339 | 277 | 214 | 194 | 213 | 154 | 251 | 193 | 264 | 199 | 252 | 199 | 228 | 220 | 200 | 217 | 165 | 248 | 167 | 231 |
| 12PHO908 | 247 | 198 | 208 | 217 | 214 | 277 | 281 | 214 | 194 | n/a | 154 | 251 | 193 | 264 | 199 | 250 | 203 | 228 | 220 | 202 | 217 | 165 | 248 | 179 | 231 |
| 13PHO427 | 247 | 211 | 207 | 217 | 214 | 341 | 277 | 214 | 194 | 213 | 154 | 253 | 193 | 264 | 199 | 252 | 199 | 228 | 220 | 200 | 217 | 165 | 248 | 167 | 231 |
| ATCC 66136 (637) | 247 | 211 | 208 | 217 | 214 | 339 | 277 | 214 | 194 | 213 | 154 | 253 | 193 | 264 | 190 | 250 | 199 | 228 | 220 | 200 | 217 | 165 | 248 | 167 | 231 |
| FR00059/2009 | 247 | 211 | 207 | 217 | 214 | 337 | 277 | 214 | 194 | 213 | 154 | 253 | 193 | 260 | 199 | 252 | 199 | 228 | 220 | 200 | 217 | 165 | 248 | 167 | 231 |
| M1384 | 247 | 211 | 207 | 217 | 214 | 275 | 277 | 214 | 194 | 213 | 154 | 251 | 193 | 264 | 199 | 252 | 199 | 228 | 220 | 200 | 217 | 165 | 248 | 167 | 231 |
| SF09937/2009 | 247 | 211 | 207 | 217 | 214 | 339 | 277 | 214 | 194 | 213 | 154 | 253 | 193 | 260 | 199 | 252 | 199 | 228 | 220 | 200 | 217 | 165 | 248 | 167 | 231 |
| 590 | 247 | 211 | 207 | 217 | 214 | 277 | 277 | 214 | 194 | 213 | 154 | 247 | 191 | 260 | 199 | 252 | 199 | 228 | 220 | 200 | 217 | 165 | 248 | 179 | 231 |
| 594 | 247 | 211 | 208 | 217 | 214 | 277 | 277 | 214 | 194 | 213 | 154 | 247 | 191 | 260 | 199 | 252 | 199 | 228 | 220 | 200 | 217 | 165 | 248 | 179 | 231 |
| 600 | 247 | 211 | 207 | 217 | 214 | 277 | 277 | 214 | 194 | 213 | 154 | 247 | 191 | 260 | 199 | 252 | 199 | 228 | 220 | 200 | 217 | 165 | 248 | 179 | 231 |
| 641 | 247 | 211 | 207 | 217 | 214 | 277 | 277 | 214 | 194 | 213 | 154 | 247 | 191 | 260 | 199 | 252 | 199 | 228 | 220 | 200 | 217 | 165 | 248 | 179 | 231 |
| 642 | 247 | 211 | 207 | 217 | 214 | 277 | 277 | 214 | 194 | 213 | 154 | 247 | 191 | 260 | 199 | 252 | 199 | 228 | 220 | 200 | 217 | 165 | 248 | 179 | 231 |
| 13PHO353 | 247 | 213 | 207 | 217 | 214 | 275 | 277 | 214 | 194 | 213 | 154 | 247 | 191 | 260 | 199 | 252 | 199 | 228 | 220 | 200 | 217 | 165 | 248 | 179 | 231 |
| ATCC 60636 | 247 | 211 | 207 | 217 | 214 | 277 | 277 | 214 | 194 | 213 | 154 | 247 | 191 | 260 | 199 | 252 | 199 | 228 | 220 | 200 | 217 | 165 | 248 | 179 | 231 |
| ATCC 62541 (601) | 247 | 211 | 207 | 217 | 214 | 277 | 277 | 214 | 194 | 213 | 154 | 247 | 191 | 260 | 199 | 252 | 199 | 228 | 220 | 200 | 217 | 165 | 248 | 179 | 231 |
| ATCC 62583 (599) | 247 | 211 | 207 | 217 | 214 | 277 | 277 | 214 | 194 | 213 | 154 | 247 | 191 | 260 | 199 | 252 | 199 | 228 | 220 | 200 | 217 | 165 | 248 | 179 | 231 |
| ATCC MYA-2585(ERC2) | 247 | 211 | 207 | 217 | 214 | 277 | 277 | 214 | 194 | 213 | 154 | 247 | 191 | 260 | 199 | 252 | 199 | 228 | 220 | 200 | 217 | 165 | 248 | 179 | 231 |
| DI 13-87 | 247 | 211 | 207 | 217 | 214 | 277 | 281 | 214 | 194 | 213 | 154 | 247 | 191 | 260 | 199 | 252 | 199 | 228 | 220 | 200 | 217 | 165 | 248 | 179 | 231 |
| SU 00AMY | 247 | 211 | 208 | 217 | 214 | 277 | 281 | 214 | 194 | 213 | 154 | 247 | 191 | 260 | 199 | 252 | 199 | 228 | 220 | 200 | 217 | 165 | 248 | 179 | 231 |

^1^nd: not determined
