# Supplementary material for: Phylogeographic Analysis of Blastomyces dermatitidis and Blastomyces gilchristii Reveals an Association with North American Freshwater Drainage Basins
Source: PLoS One. 2016 Jul 18;11(7):e0159396. doi: 10.1371/journal.pone.0159396 (PMC4948877; doi:10.1371/journal.pone.0159396)
Supplement: S2 Table — (DOCX) [file pone.0159396.s005.docx]

**S2 Table: Characteristics of *Blastomyces dermatitidis* and *Blastomyces gilchristii* isolates studied**

| **Isolate** | **Source** | **Year** | **Species** | **Pop.** | **Mating Type Allele** | **City or County** | **State/ Province** | **Latitude** | **Longitude** | **Drainage Basin** | **Reference** |
| --- | --- | --- | --- | --- | --- | --- | --- | --- | --- | --- | --- |
| ATCC 28306 | ATCC^1^ | n/a | *Bd*^2^ | 1 | HMG | n/a | Wisconsin | 44.4652 | -89.8242 | Miss^3^ | [1] |
| F2010035264 | MDH^4^ | 2010 | *Bd* | 1 | α Box | n/a | Eastern North Dakota | 47.7735 | -98.2115 | Nelson^5^ | This study |
| F2011012490 | MDH | 2011 | *Bd* | 1 | α Box | Hennepin County | Minnesota | 45.02089 | -93.5095 | Miss | This study |
| F2012014794 | MDH | 2012 | *Bd* | 1 | α Box | Koociching County | Minnesota | 48.1816 | -93.6483 | Nelson | This study |
| F2012031974 | MDH | 2012 | *Bd* | 1 | HMG | Hennepin County | Minnesota | 45.0209 | -93.5095 | Miss | This study |
| F2012034121 | MDH | 2012 | *Bd* | 1 | HMG | Ramsey County | Minnesota | 44.9964 | -93.0616 | Miss | This study |
| F2013000710 | MDH | 2013 | *Bd* | 1 | α Box | Anoka County | Minnesota | 45.3293 | -93.2197 | Miss | This study |
| M06MY001520 | AHS^6^ | 2006 | *Bd* | 1 | α Box | n/a | Alberta | 52.1906 | -113.8519 | Nelson | This study |
| M07MY006203 | AHS | 2007 | *Bd* | 1 | HMG | Regina | Saskatchewan | 50.4480 | -104.6150 | Nelson | This study |
| M07MY006625 | AHS | 2007 | *Bd* | 1 | HMG | Regina | Saskatchewan | 50.4480 | -104.6150 | Nelson | This study |
| M08MY004008 | AHS | 2008 | *Bd* | 1 | α Box | Regina | Saskatchewan | 50.4480 | -104.6150 | Nelson | This study |
| M10MY006786 | AHS | 2010 | *Bd* | 1 | HMG | Lethbridge | Alberta | 49.6935 | -112.8420 | Nelson | This study |
| M11MY004917 | AHS | 2011 | *Bd* | 1 | HMG | Saskatoon | Saskatchewan | 52.1334 | -106.6310 | Nelson | This study |
| TB00011/2006 | PHO^7^ | 2006 | *Bd* | 1 | α Box | Red Lake | Ontario | 51.0146 | -93.8289 | Nelson | [2] |
| TB00014/2008 | PHO | 2008 | *Bd* | 1 | α Box | Thunder Bay | Ontario | 48.3809 | -89.2477 | St. Law^8^ | [2] |
| TB00042/2005 | PHO | 2005 | *Bd* | 1 | HMG | Thunder Bay | Ontario | 48.3809 | -89.2477 | St. Law | [2] |
| UAMH 4042 | UAMH^9^ | 1976 | *Bd* | 1 | n/a | Regina | Saskatchewan | 50.4547 | -104.6070 | Nelson | [2] |
| UAMH 5634 | UAMH | n/a | *Bd* | 1 | HMG | Regina | Saskatchewan | 50.4480 | -104.6150 | Nelson | [2] |
| UAMH 5635 | UAMH | n/a | *Bd* | 1 | HMG | Regina | Saskatchewan | 50.4480 | -104.6150 | Nelson | [2] |
| UAMH 7800 | UAMH | 1994 | *Bd* | 1 | HMG | Calgary | Alberta | 51.0453 | -114.0580 | Nelson | [2] |
| 11PHO947 | PHO | 2011 | *Bd* | 2 | α Box | Emburn | Ontario | 45.2757 | -75.2754 | St. Law | This study |
| 11PHO646 | PHO | 2011 | *Bd* | 2 | α Box | Ottawa | Ontario | 45.4215 | -75.6972 | St. Law | This study |
| 11PHO547 | PHO | 2011 | *Bd* | 2 | HMG | Kitchner | Ontario | 43.4343 | -80.4778 | St. Law | This study |
| 12PHO296 | PHO | 2012 | *Bd* | 2 | α Box | Napanee | Ontario | 44.2481 | -76.9499 | St. Law | This study |
| 12PHO094 | PHO | 2012 | *Bd* | 2 | HMG | Barrie | Ontario | 44.3681 | -79.7176 | St. Law | This study |
| 12PHO950 | PHO | 2012 | *Bd* | 2 | n/a | King City | Ontario | 43.9287 | -79.5281 | St. Law | This study |
| 13PHO345 | PHO | 2013 | *Bd* | 2 | HMG | Ottawa | Ontario | 45.4215 | -75.6972 | St. Law | This study |
| 13PHO534 | PHO | 2013 | *Bd* | 2 | n/a | Warkworth | Ontario | 44.1994 | -77.8915 | St. Law | This study |
| 13PHO421 | PHO | 2013 | *Bd* | 2 | HMG | York | Ontario | 43.6862 | -79.4430 | St. Law | This study |
| DI 13-69 | UTFTL^10^ | n/a | *Bd* | 2 | HMG | n/a | Vermont | 44.5588 | -72.5778 | St. Law | This study |
| DI 13-71 | UTFTL | n/a | *Bd* | 2 | HMG | n/a | Vermont | 44.5588 | -72.5778 | St. Law | This study |
| DI 13-73 | UTFTL | n/a | *Bd* | 2 | HMG | n/a | Vermont | 44.5588 | -72.5778 | St. Law | This study |
| DI 13-74 | UTFTL | n/a | *Bd* | 2 | HMG | n/a | Vermont | 44.5588 | -72.5778 | St. Law | This study |
| DI 13-78 | UTFTL | n/a | *Bd* | 2 | α Box | n/a | Vermont | 44.5588 | -72.5778 | St. Law | This study |
| LSPQ-00666 | LSPQ^11^ | 2006 | *Bd* | 2 | α Box | Laval | Quebec | 45.6067 | -73.7124 | St. Law | This study |
| LSPQ-00755 | LSPQ | 2007 | *Bd* | 2 | HMG | Montreal | Quebec | 45.5087 | -73.5540 | St. Law | This study |
| LSPQ-00854 | LSPQ | 2009 | *Bd* | 2 | α Box | Saint-Augustin-de-Desmaures | Quebec | 46.7470 | -71.4593 | St. Law | This study |
| LSPQ-00859 | LSPQ | 2009 | *Bd* | 2 | α Box | Hors-Quebec | Quebec | 45.5174 | -73.5956 | St. Law | This study |
| LSPQ-00876 | LSPQ | 2009 | *Bd* | 2 | α Box | Saint-Alphonse | Quebec | 45.3131 | -72.7904 | St. Law | This study |
| LSPQ-00884 | LSPQ | 2009 | *Bd* | 2 | n/a | Saint-Charles-Borromee | Quebec | 46.0485 | -73.4627 | St. Law | This study |
| LSPQ-00941 | LSPQ | 2010 | *Bd* | 2 | HMG | Gatineau | Quebec | 45.4765 | -75.7013 | St. Law | This study |
| LSPQ-00962 | LSPQ | 2011 | *Bd* | 2 | α Box | Saint-Jerome | Quebec | 45.7754 | -74.0050 | St. Law | This study |
| LSPQ-01031 | LSPQ | 2012 | *Bd* | 2 | α Box | Saint-Jean-Chrysostome | Quebec | 46.7199 | -71.1940 | St. Law | This study |
| M1954 | WADS^12^ | 2009 | *Bd* | 2 | HMG | Schenectady | New York | 42.8142 | -73.9396 | NE Atlantic^13^ | This study |
| M2166 | WADS | 2010 | *Bd* | 2 | α Box | Kings | New York | 40.6500 | -73.9500 | NE Atlantic | This study |
| M2574 | WADS | 2010 | *Bd* | 2 | HMG | Madison | New York | 42.8990 | -75.5121 | NE Atlantic | This study |
| M3626 | WADS | 2012 | *Bd* | 2 | α Box | Montgomery | New York | 41.5276 | -74.2368 | NE Atlantic | This study |
| MYCO-01451 | LSPQ | 2005 | *Bd* | 2 | α Box | Gatineau | Quebec | 45.4765 | -75.7013 | St. Law | This study |
| SF01520/2010 | PHO | 2010 | *Bd* | 2 | α Box | Woodbridge | Ontario | 43.7891 | -79.6130 | St. Law | This study |
| SF06587/2009 | PHO | 2009 | *Bd* | 2 | HMG | Willow Beach | Ontario | 45.0553 | -79.4226 | St. Law | [2] |
| SF08339/2009 | PHO | 2009 | *Bd* | 2 | HMG | North Bay | Ontario | 46.3091 | -79.4608 | St. Law | This study |
| SF12545/2009 | PHO | 2009 | *Bd* | 2 | α Box | Parry Sound | Ontario | 45.3474 | -80.0348 | St. Law | This study |
| 371 | DA^14^ | n/a | *Bd* | 3 | α Box | n/a | South Carolina | 33.8361 | -81.1637 | SE Atlantic ^15^ | [1] |
| 663 | DA | n/a | *Bd* | 3 | α Box | Traveler's Rest | South Carolina | 34.9676 | -82.4435 | SE Atlantic | [1] |
| 664 | DA | n/a | *Bd* | 3 | α Box | Traveler's Rest | South Carolina | 34.9676 | -82.4435 | SE Atlantic | [1] |
| ATCC 26197 (GA-1) | ATCC | n/a | *Bd* | 3 | α Box | n/a | Georgia | 32.1574 | -82.9071 | SE Atlantic | [1] |
| ATCC 60916 (A2) | ATCC | n/a | *Bd* | 3 | HMG | n/a | South Carolina | 33.8361 | -81.1637 | SE Atlantic | [1] |
| DI 13-61 | UTFTL | n/a | *Bd* | 3 | α Box | n/a | North Carolina | 35.7596 | -79.0193 | SE Atlantic | This study |
| Gu | DA | n/a | *Bd* | 3 | HMG | Chicago | Illinois | 41.8781 | -87.6298 | Miss | [1] |
| K966 | DA | n/a | *Bd* | 3 | HMG | n/a | Kentucky | 37.8393 | -84.2700 | Miss | [1] |
| Ro | DA | n/a | *Bd* | 3 | α Box | n/a | Louisiana | 31.2448 | -92.1450 | Miss | [1] |
| UAB 00001 | UAB^16^ | 1994 | *Bd* | 3 | α Box | Birmingham | Alabama | 33.5207 | -86.8025 | G of Mex^17^ | This study |
| UAB 00018 | UAB | 1997 | *Bd* | 3 | HMG | Birmingham | Alabama | 33.5207 | -86.8025 | G of Mex. | This study |
| UAB 00019 | UAB | 1997 | *Bd* | 3 | HMG | Birmingham | Alabama | 33.5207 | -86.8025 | G of Mex. | This study |
| UAB 00024 | UAB | 1998 | *Bd* | 3 | α Box | Birmingham | Alabama | 33.5207 | -86.8025 | G of Mex. | This study |
| UAB 00025 | UAB | 1998 | *Bd* | 3 | n/a | Birmingham | Alabama | 33.5207 | -86.8025 | G of Mex. | This study |
| UAB 00026 | UAB | 1999 | *Bd* | 3 | HMG | Birmingham | Alabama | 33.5207 | -86.8025 | G of Mex. | This study |
| UAB 00033 | UAB | 2000 | *Bd* | 3 | HMG | Birmingham | Alabama | 33.5207 | -86.8025 | G of Mex. | This study |
| UAB 00034 | UAB | 2000 | *Bd* | 3 | HMG | Birmingham | Alabama | 33.5207 | -86.8025 | G of Mex. | This study |
| UAB 00036 | UAB | 2000 | *Bd* | 3 | HMG Box (-) | Birmingham | Alabama | 33.5207 | -86.8025 | G of Mex. | This study |
| UAB 00037 | UAB | 2000 | *Bd* | 3 | HMG | Birmingham | Alabama | 33.5207 | -86.8025 | G of Mex. | This study |
| UAB 00038 | UAB | 2000 | *Bd* | 3 | n/a | Birmingham | Alabama | 33.5207 | -86.8025 | G of Mex. | This study |
| UAB 00040 | UAB | 2000 | *Bd* | 3 | α Box | Birmingham | Alabama | 33.5207 | -86.8025 | G of Mex. | This study |
| UAB 00041 | UAB | 2000 | *Bd* | 3 | α Box | Birmingham | Alabama | 33.5207 | -86.8025 | G of Mex. | This study |
| UAB 00043 | UAB | 2000 | *Bd* | 3 | α Box | Birmingham | Alabama | 33.5207 | -86.8025 | G of Mex. | This study |
| UAB 00047 | UAB | 2001 | *Bd* | 3 | α Box | Birmingham | Alabama | 33.5207 | -86.8025 | G of Mex. | This study |
| 397 | DA | n/a | *Bd* | 4 | HMG | n/a | Georgia^18^ | 34.8582 | -84.3063 | Miss | [1] |
| 12MYC01064 | ISDH^19^ | 2012 | *Bd* | 4 | α Box | Central IN | Indiana | 40.2672 | -86.1349 | Miss | This study |
| 12MYC0354 | ISDH | 2011 | *Bd* | 4 | α Box | Central IN | Indiana | 40.2672 | -86.1349 | Miss | This study |
| 12MYC0592 | ISDH | 2011 | *Bd* | 4 | α Box | Central IN | Indiana | 40.2672 | -86.1349 | Miss | This study |
| 12MYC0593 | ISDH | 2011 | *Bd* | 4 | HMG | Central IN | Indiana | 40.2672 | -86.1349 | Miss | This study |
| 13MYC0115 | ISDH | 2012 | *Bd* | 4 | α Box | Central IN | Indiana | 40.2672 | -86.1349 | Miss | This study |
| ATCC MYA-2586 (ER-3) | ATCC | 1997 | *Bd* | 4 | HMG | Eagle River | Wisconsin^18^ | 45.9172 | -89.2443 | Miss | [3] |
| ATCC 18187 (CBS 673.68) | ATCC | n/a | *Bd* | 4 | HMG | Milwaukee | Wisconsin | 43.0389 | -87.9065 | St. Law | [4] |
| ATCC 18188 (CBS 674.68) | ATCC | n/a | *Bd* | 4 | α Box | Milwaukee | Wisconsin | 43.0389 | -87.9065 | St. Law | [4] |
| CH-10 | DA | n/a | *Bd* | 4 | α Box | n/a | Mississippi | 32.3918 | -90.4972 | Miss | [1] |
| CH-6 | DA | n/a | *Bd* | 4 | α Box | n/a | Mississippi | 32.3918 | -90.4972 | Miss | [1] |
| DI 13-101 | UTFTL | n/a | *Bd* | 4 | HMG | n/a | Wisconsin | 44.4156 | -89.8242 | Miss | This study |
| DI 13-12 | UTFTL | n/a | *Bd* | 4 | HMG | n/a | Illinois | 40.6331 | -89.3985 | Miss | This study |
| DI 13-15 | UTFTL | n/a | *Bd* | 4 | HMG | n/a | Illinois | 40.6331 | -89.3985 | Miss | This study |
| DI 13-18 | UTFTL | n/a | *Bd* | 4 | Α Box | n/a | Illinois | 40.6331 | -89.3985 | Miss | This study |
| DI 13-20 | UTFTL | n/a | *Bd* | 4 | HMG | n/a | Illinois | 40.6331 | -89.3985 | Miss | This study |
| DI 13-21 | UTFTL | n/a | *Bd* | 4 | HMG | n/a | Illinois | 40.6331 | -89.3985 | Miss | This study |
| DI 13-28 | UTFTL | n/a | *Bd* | 4 | Α Box | n/a | Illinois | 40.6331 | -89.3985 | Miss | This study |
| DI 13-30 | UTFTL | n/a | *Bd* | 4 | Α Box | n/a | Illinois | 40.6331 | -89.3985 | Miss | This study |
| DI 13-36 | UTFTL | n/a | *Bd* | 4 | HMG | n/a | Arkansas | 35.2011 | -91.8318 | Miss | This study |
| DI 13-37 | UTFTL | n/a | *Bd* | 4 | Α Box | n/a | Arkansas | 35.2011 | -91.8318 | Miss | This study |
| DI 13-57 | UTFTL | n/a | *Bd* | 4 | HMG | n/a | Missouri | 37.9643 | -91.8318 | Miss | This study |
| DI 13-58 | UTFTL | n/a | *Bd* | 4 | HMG | n/a | Missouri | 37.9643 | -91.8318 | Miss | This study |
| DI 13-60 | UTFTL | n/a | *Bd* | 4 | Α Box | n/a | Missouri | 37.9643 | -91.8318 | Miss | This study |
| DI 13-63 | UTFTL | n/a | *Bd* | 4 | HMG | n/a | Ohio | 40.4173 | -82.9071 | Miss | This study |
| DI 13-64 | UTFTL | n/a | *Bd* | 4 | HMG | n/a | Texas | 33.5209 | -95.1557 | Miss | This study |
| DI 13-66 | UTFTL | n/a | *Bd* | 4 | HMG | n/a | Texas | 33.5209 | -95.1557 | Miss | This study |
| DI 13-67 | UTFTL | n/a | *Bd* | 4 | HMG | n/a | Texas | 33.5209 | -95.1557 | Miss | This study |
| DI 13-80 | UTFTL | n/a | *Bd* | 4 | Α Box | n/a | Wisconsin | 44.4652 | -89.8242 | Miss | This study |
| DI 13-81 | UTFTL | n/a | *Bd* | 4 | Α Box | n/a | Wisconsin | 44.4652 | -89.8242 | Miss | This study |
| DI 13-83 | UTFTL | n/a | *Bd* | 4 | HMG | n/a | Wisconsin | 44.4652 | -89.8242 | Miss | This study |
| DI 13-86 | UTFTL | n/a | *Bd* | 4 | HMG | n/a | Wisconsin | 44.4652 | -89.8242 | Miss | This study |
| DI 13-9 | UTFTL | n/a | *Bd* | 4 | Α Box | n/a | Illinois | 40.6331 | -89.3985 | Miss | This study |
| DI 13-96 | UTFTL | n/a | *Bd* | 4 | α Box | n/a | Wisconsin | 44.4652 | -89.8242 | Miss | This study |
| En | DA | n/a | *Bd* | 4 | HMG | Chicago | Illinois | 41.87811 | -87.6298 | Miss | [1] |
| F2011027401 | MDH | 2011 | *Bd* | 4 | HMG | Ramsey County | Minnesota | 44.9964 | -93.0616 | Miss | This study |
| MICH-1 | MDCH^20^ | 2012 | *Bd* | 4 | α Box | Madison Heights | Michigan | 42.4859 | -83.1052 | St. Law | This study |
| MICH-2 | MDCH | 2012 | *Bd* | 4 | α Box | Brimley | Michigan | 46.4041 | -84.5723 | St. Law | This study |
| MICH-3 | MDCH | 2012 | *Bd* | 4 | α Box | n/a | Michigan | 44.3148 | -85.6024 | St. Law | This study |
| SACR | DA | n/a | *Bd* | 4 | α Box | n/a | n/a | n/a | n/a | n/a | [5] |
| SF06266/2009 | PHO | 2009 | *Bd* | 4 | HMG | Mississauga | Ontario | 43.5891 | -79.6441 | St. Law | [2] |
| SU-SACS | DA | n/a | *Bd* | 4 | α Box | n/a | n/a | n/a | n/a | n/a | [5] |
| UAMH 5584 | UAMH | 1986 | *Bd* | 4 | HMG | Edmonton | Alberta^21^ | 53.5444 | -113.4910 | Nelson | [2] |
| 12PHO936 | PHO | 2012 | *Bg*^22^ | 1 | HMG | Sault Ste Marie | Ontario | 46.5128 | -84.2703 | St. Law | This study |
| 12PHO819 | PHO | n/a | *Bg* | 1 | n/a | Mine Centre | Ontario | 48.9238 | -92.9168 | Nelson | This study |
| 12PHO858 | PHO | 2012 | *Bg* | 1 | α Box | Sault Ste Marie | Ontario | 46.5128 | -84.2703 | St. Law | This study |
| 12PHO859 | PHO | n/a | *Bg* | 1 | α Box | Fort Frances | Ontario | 48.6100 | -93.3955 | Nelson | This study |
| F2011036969 | MDH | 2011 | *Bg* | 1 | HMG | Hennepin County | Minnesota | 45.0209 | -93.5095 | Miss | This study |
| F2013001127 | MDH | 2013 | *Bg* | 1 | α Box | St. Louis County | Minnesota | 47.7395 | -92.3624 | Nelson | This study |
| M08MY007367 | AHS | 2008 | *Bg* | 1 | HMG | Edmonton | Alberta | 53.5444 | -113.4910 | Nelson | This study |
| M09MY002766 | AHS | 2009 | *Bg* | 1 | α Box | Saskatoon | Saskatchewan | 52.1344 | -106.6480 | Nelson | This study |
| M10MY005485 | AHS | 2010 | *Bg* | 1 | α Box | Vancouver | British Columbia | 49.2612 | -123.1140 | Fraser^23^ | This study |
| M11MY006516 | AHS | 2011 | *Bg* | 1 | α Box | Edmonton | Alberta | 53.5444 | -113.4910 | Nelson | This study |
| TB00002/2008 | PHO | 2008 | *Bg* | 1 | HMG | Kenora | Ontario | 49.7670 | -94.4894 | Nelson | [2] |
| TB00016/2005 | PHO | 2005 | *Bg* | 1 | HMG | Shoal Lake | Ontario | 49.6180 | -95.1082 | Nelson | [2] |
| TB00017/2006 | PHO | 2006 | *Bg* | 1 | α Box | Kenora | Ontario | 49.7670 | -94.4894 | Nelson | [2] |
| TB00018/2005 | PHO | 2005 | *Bg* | 1 | α Box | Kenora | Ontario | 49.7670 | -94.4894 | Nelson | [2] |
| TB00018/2006 | PHO | 2006 | *Bg* | 1 | HMG | Keewatin | Ontario | 49.7621 | -94.5538 | Nelson | [2] |
| TB00019/2005 | PHO | 2005 | *Bg* | 1 | HMG | Kenora | Ontario | 49.7670 | -94.4894 | Nelson | [2] |
| TB00022/2006 | PHO | 2006 | *Bg* | 1 | HMG | Kenora | Ontario | 49.7670 | -94.4894 | Nelson | [2] |
| TB00023/2005 | PHO | 2005 | *Bg* | 1 | HMG | Kenora | Ontario | 49.7670 | -94.4894 | Nelson | [2] |
| TB00025/2005 | PHO | 2005 | *Bg* | 1 | α Box | Kenora | Ontario | 49.7670 | -94.4894 | Nelson | [2] |
| TB00029/2006 | PHO | 2006 | *Bg* | 1 | α Box | Kenora | Ontario | 49.7670 | -94.4894 | Nelson | [2] |
| TB00032/2006 | PHO | 2006 | *Bg* | 1 | HMG | North Spirit Lake | Ontario | 52.5069 | -93.0237 | Hudson^24^ | [2] |
| TB00037/2008 | PHO | 2008 | *Bg* | 1 | HMG | Keewatin | Ontario | 49.7621 | -94.5538 | Nelson | [2] |
| TB00038/2005 | PHO | 2005 | *Bg* | 1 | HMG | Morson | Ontario | 49.0978 | -94.3152 | Nelson | [2] |
| TB00040/2005 | PHO | 2005 | *Bg* | 1 | α Box | Sioux Lookout | Ontario | 50.09783 | -91.9220 | Nelson | [2] |
| F2010034629 | MDH | 2010 | *Bg* | 2 | α Box | n/a | Western Wisconsin | 44.44162 | -91.5491 | Miss | This study |
| F2010037690 | MDH | 2010 | *Bg* | 2 | HMG | Hennepin County | Minnesota | 45.0209 | -93.5095 | Miss | This study |
| F2011026044 | MDH | 2011 | *Bg* | 2 | HMG | Ramsey County | Minnesota | 44.9964 | -93.0616 | Miss | This study |
| F252 | DA | n/a | *Bg* | 2 | α Box | n/a | Wisconsin | 44.4652 | -89.8242 | Miss | [1] |
| 10PHO703 | PHO | 2010 | *Bg* | 3 | HMG | Toronto | Ontario | 43.6532 | -79.3832 | St. Law | This study |
| 10PHO673 | PHO | 2010 | *Bg* | 3 | HMG | Forest | Ontario | 44.3682 | -76.6182 | St. Law | This study |
| 11PHO185 | PHO | 2011 | *Bg* | 3 | HMG | Oakville | Ontario | 43.4675 | -79.6877 | St. Law | This study |
| 12PHO241 | PHO | 2012 | *Bg* | 3 | n/a | Mississauga | Ontario | 43.5881 | -79.7512 | St. Law | This study |
| 12PHO908 | PHO | 2012 | *Bg* | 3 | α Box | Sudbury | Ontario | 46.5199 | -80.9437 | St. Law | This study |
| 13PHO427 | PHO | 2013 | *Bg* | 3 | α Box | Kitchner | Ontario | 43.4343 | -80.4778 | St. Law | This study |
| ATCC 66136 (637) | ATCC | 1986 | *Bg* | 3 | α Box | Sarnia | Ontario^18^ | 42.9745 | -82.4066 | St. Law | [6] |
| FR00059/2009 | PHO | 2009 | *Bg* | 3 | HMG | Little Current | Ontario | 45.9805 | -81.9278 | St. Law | [2] |
| M1384 | WADS | 2008 | *Bg* | 3 | HMG | Monrose | New York | 41.2524 | -73.9319 | NE Atlantic | This study |
| SF09937/2009 | PHO | n/a | *Bg* | 3 | HMG | Sudbury | Ontario | 46.4900 | -81.0100 | St. Law | This study |
| 590 | DA | n/a | *Bg* | 4 | HMG | Eagle River | Wisconsin | 45.9172 | -89.2443 | Miss | [1] |
| 594 | DA | n/a | *Bg* | 4 | α Box | Eagle River | Wisconsin | 45.9172 | -89.2443 | Miss | [1] |
| 600 | DA | n/a | *Bg* | 4 | HMG | Eagle River | Wisconsin | 45.9172 | -89.2443 | Miss | [1] |
| 641 | DA | n/a | *Bg* | 4 | HMG | Oconto Falls | Wisconsin | 44.8739 | -88.1429 | St. Law | [1] |
| 642 | DA | n/a | *Bg* | 4 | HMG | Oconto Falls | Wisconsin | 44.8739 | -88.1429 | St. Law | [1] |
| 13PHO353 | PHO | 2013 | *Bg* | 4 | HMG | Toronto | Ontario | 43.6532 | -79.3832 | St. Law | This study |
| ATCC 60636 | ATCC | 1984 | *Bg* | 4 | HMG | Eagle River | Wisconsin^18^ | 45.9172 | -89.2443 | Miss | [7] |
| ATCC 62541 (601) | ATCC | 1985 | *Bg* | 4 | α Box | Eagle River | Wisconsin | 45.9172 | -89.2443 | Miss | [7] |
| ATCC 62583 (599) | ATCC | 1985 | *Bg* | 4 | HMG | Eagle River | Wisconsin | 45.9172 | -89.2443 | Miss | [7] |
| ATCC MYA-2585 (ERC-2) | ATCC | 1996 | *Bg* | 4 | HMG | Eagle River | Wisconsin | 45.9172 | -89.2443 | Miss | [8] |
| DI 13-87 | UTFTL | n/a | *Bg* | 4 | HMG | n/a | Wisconsin | 44.4652 | -89.8242 | Miss | This study |
| SU 00AMY | DA | n/a | *Bg* | 4 | HMG | Tomorrow River | Wisconsin | 44.5553 | -89.3571 | St. Law | [1] |

^1^ATCC: America Type Culture Collection (Manassa, VA, USA)

^2^*Bd*: *Blastomyces dermatitidis*

^3^Miss: Mississippi River drainage basin

^4^MDH: Minnesota Department of Health (St. Paul, MN, USA)

^5^Nelson: Nelson River drainage basin

^6^AHS: Alberta Health Services, University of Alberta Hospital (Edmonton, AB, Canada)

^7^PHO: Mycology laboratory at Public Health Ontario (Toronto, ON, Canada)

^8^St. Law: St. Lawrence River drainage basin

^9^UAMH: University of Alberta Microfungus and Herbarium (Edmonton, AB, Canada)

^10^UTFTL: University of Texas Fungus Testing Laboratory (San Antonio, TX, USA)

^11^LSPQ: Laboratoire de santé publique du Québec (Sainte-Anne-de-Bellevue, Québec, Canada).

^12^WADS: Wadsworth Center (Albany, NY, USA)

^13^NE Atlantic: Northeast Atlantic Ocean Seaboard drainage basin

^14^DA: David Stephens, Santa Clara Valley Medical Center (San Jose, CA, USA)

**^15^**SE Atlantic: Southeast Atlantic Ocean Seaboard drainage basin

^16^UAB: University of Alabama (Birmingham, Alabama)

^17^G of Mex.: Gulf of Mexico drainage basin

^18^Environmental isolate

^19^ISDH: Indiana State Department of Health (Indianapolis, IN, USA)

^20^MDCH: Michigan Department of Community Health (Lansing MI, USA)

^21^Canine isolate

^22^*Bg*: *Blastomyces gilchristii*

^23^Fraser: Fraser River drainage basin

^24^Hudson: Hudson Bay drainage basin

**References**

1. McCullough MJ, DiSalvo AF, Clemons KV, Park P, Stevens DA. Molecular epidemiology of *Blastomyces* *dermatitidis*. Clin Infect Dis. 2000;30: 328-335.

2. Brown EM, McTaggart LR, Zhang SX, Low DE, Stevens DA, Richardson SE. Phylogenetic analysis reveals a cryptic species *Blastomyces* *gilchristii*, sp. nov. within the human pathogenic fungus *Blastomyces* *dermatitidis*. PLoS One. 2013;8: e59237.

3. Baumgardner DJ, Paretsky DP. The in vitro isolation of *Blastomyces* *dermatitidis* from a woodpile in north central Wisconsin, USA. Med Mycol. 1999;37: 163-168.

4. Meece JK, Anderson JL, Fisher MC, Henk DA, Sloss BL, Reed KD. Population genetic structure of clinical and environmental isolates of *Blastomyces* *dermatitidis*, based on 27 polymorphic microsatellite markers. Appl Environ Microbiol. 2011;77: 5123-5131.

5. Harvey RP, Schmid ES, Carrington CC, Stevens DA. Mouse model of pulmonary blastomycosis: utility, simplicity, and quantitative parameters. Am Rev Respir Dis. 1978;117: 695-703.

6. Bakerspigel A, Kane J, Schaus D. Isolation of *Blastomyces* *dermatitidis* from an earthen floor in southwestern Ontario, Canada. J Clin Microbiol. 1986;24: 890-891.

7. Klein BS, Vergeront JM, Weeks RJ, Kumar UN, Mathai G, Varkey B, et al. Isolation of *Blastomyces* *dermatitidis* in soil associated with a large outbreak of blastomycosis in Wisconsin. N Engl J Med. 1986;314: 529-534.

8. Baumgardner DJ, Paretsky DP. Identification of *Blastomyces* *dermatitidis* in the stool of a dog with acute pulmonary blastomycosis. J Med Vet Mycol. 1997;35: 419-421.
